# Supplementary material for: Neonatal morbidity after fetal exposure to antipsychotics: a national register-based study
Source: BMJ Open. 2022 Jun 29;12(6):e061328. doi: 10.1136/bmjopen-2022-061328 (PMC9244682; doi:10.1136/bmjopen-2022-061328)
Supplement: Supplementary data [file bmjopen-2022-061328supp001.pdf]

# Supplemental material for: Heinonen et al. Neonatal morbidity after fetal exposure to antipsychotics - a national register-based study

| EXPOSURE | n |
|---|---|
| **First-generation antipsychotics** | |
| Levomepromazine | 251 |
| Flupentixol | 165 |
| Haloperidol | 162 |
| Perphenazine | 93 |
| Zuclopenthixol | 75 |
| Chlorprothixene | 45 |
| Chlorpromazine | 4 |
| Pimozide | 4 |
| Fluphenazine | 3 |
| Thioridazine | 1 |
| **Second-generation antipsychotics** | |
| Quetiapine | 1026 |
| Olanzapine | 771 |
| Aripiprazole | 334 |
| Risperidone | 191 |
| Ziprasidone | 34 |
| Clozapine | 29 |
| Paliperidone | 9 |
| Sertrindole | 2 |
| **TOTAL** | 3199 |

**Supplemental Table 1.** Prescriptions of antipsychotics during pregnancy 2006-2017.

| NICU-admissions | Exposed vs Never exposed | | | | | | Exposed Infants vs Infants to Mothers Treated with Antipsychotics Before or After the Pregnancy | | | | | |
|---|---|---|---|---|---|---|---|---|---|---|---|---|
| | Crude | | Adjusted for Maternal Factors Only | | Adjusted for Maternal and Fetal Factors | | Crude | | Adjusted for Maternal Factors Only | | Adjusted for Maternal and Fetal Factors | |
| | RR | 95% CI | RR | 95% CI | RR | 95% CI | RR | 95% CI | RR | 95% CI | RR | 95% CI |
| **Exposed early pregnancy only** | | | | | | | | | | | | |
| Any Antipsychotics, n=1454 | 2.2 | 2.0-2.5 | 1.5 | 1.3-1.7 | 1.3 | 1.1-1.6 | 1.5 | 1.3-1.7 | 1.3 | 1.1-1.4 | 1.2 | 1.0-1.4 |
| F-GA, n=431 | 2.2 | 1.7-2.7 | 1.5 | 1.2-1.9 | 1.5 | 1.2-1.8 | 1.5 | 1.2-1.8 | 1.3 | 1.0-1.6 | 1.3 | 1.0-1.6* |
| S-GA, n=1166 | 2.3 | 2.0-2.6 | 1.5 | 1.3-1.7 | 1.3 | 1.0-1.7* | 1.5 | 1.3-1.7 | 1.3 | 1.1-1.4 | 1.2 | 0.9-1.4 |
| **Exposed in late pregnancy** | | | | | | | | | | | | |
| Any Antipsychotics, n=1223 | 2.8 | 2.5-3.1 | 1.8 | 1.6-2.0 | 1.8 | 1.6-2.0 | 1.9 | 1.7-2.1 | 1.5 | 1.4-1.7 | 1.5 | 1.4-1.7 |
| F-GA, n=297 | 3.1 | 2.6-3.8 | 2.1 | 1.7-2.5 | 2.1 | 1.7-2.5 | 2.1 | 1.7-2.6 | 1.7 | 1.4-2.1 | 1.7 | 1.4-2.0 |
| S-GA, n=972 | 2.7 | 2.4-3.1 | 1.8 | 1.6-2.0 | 1.8 | 1.6-2.0 | 1.9 | 1.6-2.1 | 1.5 | 1.3-1.7 | 1.5 | 1.3-1.7 |
| **Never exposed, n=1262 047** | REFERENCE GROUP 1 | | | | | | | | | | | |
| **Exposed before or after but not during pregnancy, n=34 492** | REFERENCE GROUP 2 | | | | | | | | | | | |

**Supplemental table 2.** Risk (RR) for admission to neonatal intensive care unit (NICU) compared with non-exposed infants and with infants to mothers using antipsychotics before and/or after but not during the current pregnancy. F-GA = First-generation Antipsychotics, S-GA = Second-generation Antipsychotics. Maternal factors: Primipara, Age, Body Mass Index (BMI), Smoking, Cesarean section, Concurrent neurotropic drugs. Fetal factors: Gestational Age, Z-score. * p <0.05

| Neonatal outcomes | Exposed vs Never exposed | | | | | | Exposed Infants vs Infants to Mothers Treated with Antipsychotics Before or After the Pregnancy | | | | | |
|---|---|---|---|---|---|---|---|---|---|---|---|---|
| | Crude | | Adjusted for Maternal Factors Only | | Adjusted for Maternal and Fetal Factors | | Crude | | Adjusted for Maternal Factors Only | | Adjusted for Maternal and Fetal Factors | |
| | RR | 95% CI | RR | 95% CI | RR | 95% CI | RR | 95% CI | RR | 95% CI | RR | 95% CI |
| **Admission to NICU** | 2.5 | 2.3-2.7 | 1.7 | 1.6-1.8 | 1.6 | 1.4-1.6 | 1.7 | 1.5-1.8 | 1.4 | 1.3-1.5 | 1.4 | 1.2-1.5 |
| **Respiratory symptoms** | | | | | | | | | | | | |
| Transient tachypnea of the newborn | 2.5 | 2.2-2.9 | 1.6 | 1.4-1.9 | 1.5 | 1.3-1.8 | 1.7 | 1.5-2.0 | 1.4 | 1.2-1.7 | 1.4 | 1.2-1.6 |
| Persistent pulmonary hypertension | 2.8 | 1.9-4.1 | 2.1 | 1.4-3.1 | 1.5 | 0.9-2.6 | 1.7 | 1.1-2.5 | 1.5 | 1.0-2.2 | 1.2 | 0.7-2.2 |
| Respiratory distress syndrome | 1.5 | 1.0-2.3 | 1.0 | 0.7-1.6 | 0.7 | 0.3-1.5 | 0.9 | 0.6-1.4 | 0.9 | 0.6-1.4 | 0.6 | 0.3-1.3 |
| **Respiratory treatment** | | | | | | | | | | | | |
| CPAP | 2.7 | 2.3-3.1 | 1.7 | 1.4-2.0 | 1.5 | 1.1-2.0 | 1.7 | 1.5-2.0 | 1.5 | 1.2-1.7 | 1.3 | 1.0-1.7 |
| Ventilator treatment | 1.5 | 1.0-2.3 | 1.1 | 0.7-1.6 | 0.8 | 0.4-1.5 | 1.0 | 0.6-1.5 | 0.9 | 0.6-1.4 | 0.7 | 0.3-1.4 |
| **Hyperbilirubinemia** | 1.5 | 1.3-1.7 | 1.3 | 1.1-1.5 | 1.2 | 0.9-1.4 | 1.2 | 1.0-1.4* | 1.1 | 1.0-1.3 | 1.1 | 0.9-1.3 |
| **Hypoglycemia** | 2.2 | 1.9-2.6 | 1.4 | 1.2-1.7 | 1.3 | 1.1-1.6 | 1.6 | 1.3-1.9 | 1.3 | 1.1-1.5 | 1.2 | 1.0-1.5* |
| **Feeding difficulties** | 2.5 | 2.0-3.1 | 1.8 | 1.4-2.3 | 1.7 | 1.3-2.2 | 1.7 | 1.4-2.2 | 1.6 | 1.2-2.0 | 1.5 | 1.1-1.9 |
| **Neurological disorders** | 5.8 | 4.4-7.7 | 3.4 | 2.5-4.7 | 3.3 | 2.4-4.6 | 3.3 | 2.4-4.5 | 2.3 | 1.6-3.3 | 2.3 | 1.6-3.3 |
| **Withdrawal symptoms from therapeutic drugs** | 105 | 72.4-152 | 17.7 | 9.6-32.6 | 17.3 | 9.4-31.7 | 5.3 | 3.6-8.0 | 3.3 | 2.1-5.3 | 3.4 | 2.1-5.3 |

**Supplemental table 3.** Risk (RR) for neonatal morbidity after exposure to antipsychotics. NICU = Neonatal intensive Care Unit. CPAP = Continuous positive airway pressure. Maternal factors: Primipara, Age, Body Mass Index (BMI), Smoking, Cesarean section, Concurrent neurotropic drugs. Fetal factors: Gestational Age, Z-score. * p <0.05
