# Supplementary material for: Neonatal morbidity after fetal exposure to antipsychotics: a national register-based study
Source: BMJ Open. 2022 Jun 29;12(6):e061328. doi: 10.1136/bmjopen-2022-061328 (PMC9244682; doi:10.1136/bmjopen-2022-061328)
Supplement: Author's manuscript [file bmjopen-2022-061328.draft_revisions.pdf]

BMJ Open is committed to open peer review. As part of this commitment we make the peer review history of every article we publish publicly available.

When an article is published we post the peer reviewers' comments and the authors' responses online. We also post the versions of the paper that were used during peer review. These are the versions that the peer review comments apply to.

The versions of the paper that follow are the versions that were submitted during the peer review process. They are not the versions of record or the final published versions. They should not be cited or distributed as the published version of this manuscript.

BMJ Open is an open access journal and the full, final, typeset and author-corrected version of record of the manuscript is available on our site with no access controls, subscription charges or pay-per-view fees (http://bmjopen.bmj.com).

If you have any questions on BMJ Open's open peer review process please email info.bmjopen@bmj.com

BMJ Open

Neonatal morbidity after fetal exposure to antipsychotics - a national register-based study

Journal:	*BMJ Open*
Manuscript ID	bmjopen-2022-061328
Article Type:	Original research
Date Submitted by the Author:	24-Jan-2022

Complete List of Authors:	Heinonen, Essi; Karolinska institutet Department of Clinical Sciences Intervention and Technology, Pediatrics; Karolinska University Hospital, Department of Neonatology<br>Forsberg, Lisa; Karolinska institutet Department of Clinical Sciences Intervention and Technology, Pediatrics<br>Nörby, Ulrika ; Lund University, Department of Clinical Sciences, Centre of Reproduction Epidemiology, Tornblad Institute; Region Stockholm, Health and Medical Care Administration<br>Wide, Katarina; Karolinska Institute Department of Clinical Science Intervention and Technology, Paediatrics; Karolinska Universitetssjukhuset, Paediatrics<br>Källén, Karin; Lunds Universitet, Department of Clinical Sciences, Centre of Reproduction Epidemiology, Tornblad Institute

Keywords:	CLINICAL PHARMACOLOGY, EPIDEMIOLOGY, NEONATOLOGY, Maternal medicine < OBSTETRICS, Schizophrenia & psychotic disorders < PSYCHIATRY, Depression & mood disorders < PSYCHIATRY

SCHOLARONE™
Manuscripts

I, the Submitting Author has the right to grant and does grant on behalf of all authors of the Work (as defined in the below author licence), an exclusive licence and/or a non-exclusive licence for contributions from authors who are: i) UK Crown employees; ii) where BMJ has agreed a CC-BY licence shall apply, and/or iii) in accordance with the terms applicable for US Federal Government officers or employees acting as part of their official duties; on a worldwide, perpetual, irrevocable, royalty-free basis to BMJ Publishing Group Ltd ("BMJ") its licensees and where the relevant Journal is co-owned by BMJ to the co-owners of the Journal, to publish the Work in this journal and any other BMJ products and to exploit all rights, as set out in our licence.

The Submitting Author accepts and understands that any supply made under these terms is made by BMJ to the Submitting Author unless you are acting as an employee on behalf of your employer or a postgraduate student of an affiliated institution which is paying any applicable article publishing charge ("APC") for Open Access articles. Where the Submitting Author wishes to make the Work available on an Open Access basis (and intends to pay the relevant APC), the terms of reuse of such Open Access shall be governed by a Creative Commons licence – details of these licences and which Creative Commons licence will apply to this Work are set out in our licence referred to above.

Other than as permitted in any relevant BMJ Author's Self Archiving Policies, I confirm this Work has not been accepted for publication elsewhere, is not being considered for publication elsewhere and does not duplicate material already published. I confirm all authors consent to publication of this Work and authorise the granting of this licence.

For peer review only

Neonatal morbidity after fetal exposure to antipsychotics - a national register-based study

***Essi Heinonen, MD,*[a,b] Lisa Forsberg, MD, PhD,[a] *Ulrika Nörby,* MSc Pharm, PhD,[d,e] *Katarina Wide, MD, PhD,*[a,c] *Karin Källén, Prof.*[d]**

[a] Division of Pediatrics, Department of Clinical Science, Intervention and Technology (CLINTEC), Karolinska Institutet, Stockholm, Sweden; [b] *Department of Pediatrics and Newborn medicine at Karolinska University Hospital, Stockholm, Sweden;* [c] *Department of Pediatrics and Emergency Pediatrics at Karolinska University Hospital, Stockholm, Sweden;* [d] Centre of Reproduction Epidemiology, Tornblad Institute, Department of Clinical Sciences, Lund University, Lund, Sweden; [e] Health and Medical Care Administration, Region Stockholm, Stockholm, Sweden.

Address correspondence to: Essi Heinonen, Division of Pediatrics, Department of Clinical Science, Intervention and Technology (CLINTEC), Karolinska Institutet, Stockholm, Sweden. Blickagången 6A, 14157 Huddinge, Sweden. [essi.heinonen@ki.se], +4673-563 3330.

For peer review only

For peer review only - http://bmjopen.bmj.com/site/about/guidelines.xhtml

Abstract

Objective: To investigate the admission rate to neonatal care and neonatal morbidity after maternal use of antipsychotic drugs during pregnancy.

Design: A population-based register study.

Setting: Information on all singleton births between July 2006 and December 2017 in Sweden as well as data on prescription drugs, deliveries and infants' health were obtained from the Swedish Medical Birth Register, the Prescribed Drug Register, and the Swedish Neonatal Quality Register. Exposed infants were compared to the population and to infants to mothers treated with antipsychotics before or after but not during pregnancy.

Participants: The cohort comprised a total of 1 307 487 infants, of whom 2677 (0.2%) were exposed to antipsychotics during pregnancy and 34 492 (2.6%) had mothers that were treated before and/or after pregnancy.

Outcome measures: The primary outcome was admission rate to neonatal care. Secondary outcomes were the separate neonatal morbidities available from the registers.

Results: Of the exposed infants, 19.3% were admitted to neonatal care compared with 7.8% in the population (adjusted risk ratio [aRR]: 1.7; 95% confidence interval [CI]: 1.6-1.8), with a further increased risk after exposure in late pregnancy. The highest relative risks were seen for withdrawal symptoms (aRR: 17.7; 95% CI: 9.6-32.6], neurological disorders (aRR: 3.4; 95% CI: 2.4-5.7) and persistent pulmonary hypertension, aRR: 2.1; 95% CI:1.4-3.1) when compared to the population. The absolute risks for these outcomes were however low. The relative risks were lower when exposure during pregnancy was compared to treatment before/after pregnancy.

Conclusion: Fetal exposure to antipsychotics was associated with an increased risk for neonatal care. This study indicates that drug exposure may be one of the several factors behind neonatal morbidity. The increased risk for persistent pulmonary hypertension needs to be studied further.

Article summary

Strengths and limitations of this study

- To our knowledge, this is the largest study to date to investigate the risk for individual neonatal morbidities amongst infants exposed to antipsychotics.
- The Swedish health registers have the advantage of almost complete coverage of the inhabitants, information on important confounders and minimal bias in data collection, with the Swedish Neonatal Quality Register containing detailed information on all infants admitted to neonatal care.
- We included a control group of infants to mothers treated with antipsychotics before or after but not during pregnancy that allowed us to adjust for factors associated to the underlying disorder, at least to some extent.
- Even though the study included 1.3 million births, the numbers were too low to obtain reliable risk estimates for specific antipsychotics. Instead, the analyses were limited to antipsychotics on group level.

For peer review only - http://bmjopen.bmj.com/site/about/guidelines.xhtml

Introduction

Use of antipsychotic drugs is increasing globally due to the widened indications and off-label use of second generation antipsychotics in treatment of a wide range of psychiatric disorders, also during pregnancy.[1-6] Treatment during pregnancy does not generally seem to increase the risk for malformations.[7-12] Some studies have indicated, that schizophrenia itself might be connected to an increased risk for malformations, and discontinuation of drug therapy is not recommended during pregnancy due to the risk of relapse of the underlying condition. [13-18]

Fetal exposure to antipsychotics has in smaller cohorts been linked to increased risks for prematurity, altered fetal growth, increased admission to neonatal intensive care unit (NICU), low APGAR-scores, and neonatal extrapyramidal and respiratory symptoms. These risks seem to increase with increased dosage and with polypharmacy. Several of the previous studies have not adjusted the risks for any confounding maternal factors.[9 12 16 19-21] Three studies, that adjusted for health- and lifestyle confounders in the analyses, did not confirm the increased neonatal morbidity after intrauterine antipsychotic exposure. Instead, one of them found a correlation between maternal psychiatric care during pregnancy and infant admission to NICU.[22-24] Maternal schizophrenia and bipolar disorders have independent of drug exposure been associated with increased risks of prematurity, preeclampsia, neonatal morbidity including poor arousal and potential adverse neurodevelopmental effects in the infants.[3 14 25-27] Mothers with untreated psychiatric illness are also more likely to engage in high-risk behaviours such as smoking, alcohol and illicit drugs and are less likely to attend to antenatal care.[25]

The aim of this Swedish population based register study was to clarify the pattern and the frequency of the neonatal effects associated with intrauterine antipsychotic exposure. We also attempted to separate these effects from the impact of the underlying disease, its associated risk factors and other medications.

For peer review only - http://bmjopen.bmj.com/site/about/guidelines.xhtml

Methods

The study was a register-based study combining data from the Swedish Medical Birth Register (MBR),[28] the Prescribed Drug Register (PDR),[29] The Swedish Neonatal Quality Register (SNQ),[30] and the Perinatal Revision South Register (PRS).[31] Swedish personal identification numbers were used for register linkages. The study population consisted of all singleton births in Sweden, a total of 1 307 487 infants, registered in the MBR between July 1, 2006, And December 31, 2017. An outline of the study design is presented in Figure 1.

The MBR holds data on antenatal care, delivery, and examination of the newborn for >97% of all births. Information on body mass index (BMI), medications and smoking habits are registered in the MBR at the first visit of the complimentary antenatal care offered all pregnant women in Sweden.[28] For this study, information on drug exposure and maternal and fetal background characteristics were collected from the MBR. Women with a diagnosis of pre-pregnancy diabetes or with valproate treatment (N03A G01) were excluded from the analysis. Valproate exposed infants were excluded due to the well-known teratogenicity of the drug as well as co-medication with valproate and antipsychotics being fairly common.[32]

Data on exposure for prescription drugs were also acquired from the PDR. The PDR stores data on all drugs prescribed in ambulatory care and dispensed at a Swedish pharmacy but does not include medications used in hospitals.[29] The drugs registered in the MBR and PDR are classified according to the Anatomical Therapeutic Chemical (ATC) classification system. Antipsychotic exposure was defined as drugs belonging to ATC-class N05A, antipsychotics. Exposures to the antipsychotics dixyrazine (N05AB01), prochlorperazine (N05AB04), melperone (N05AD03) and lithium (N05A N01) were excluded from the exposed group and considered as covariates, due to the use of these drugs as antiemetics (dixyrazine,

For peer review only - http://bmjopen.bmj.com/site/about/guidelines.xhtml

prochlorperazine, melperone) and as a mood stabilizer (lithium) rather than antipsychotics. The remaining drugs in class N05A were divided into first- and second-generation antipsychotics according to Supplemental Table 1.

Antipsychotics exposure was allocated into any exposure (drugs dispensed at any time during or 1 month before the pregnancy), late exposure (drugs dispensed during the last 90 days of the pregnancy with or without earlier dispenses), and early exposure only (drugs dispensed 1 month before and during pregnancy but not during the last 90 days of the pregnancy). We also created a reference group with women exposed to antipsychotics any time during the study period, before or after the pregnancy, but not during the pregnancy or 1 month before it. This group was used to attempt to control for the effect of the underlying psychiatric illness and other psychosocial factors connected to the neonatal outcome. Exposure data was also collected on the following neurotropic drugs known to or suspected to cause similar neonatal morbidity as antipsychotics: antidepressants (ATC-code N06A), antiepileptics (N03A), opioids (N02A), centrally acting sympathomimetics (N06BA), sedatives (N05B, N05C) and milder sedatives (alimemazine, promethazine and the excluded antiemetic antipsychotics from N05A).

Data on NICU admissions and neonatal outcomes were extracted from the SNQ that covers all 37 Swedish NICUs. [30] Because the south of Sweden was not included in the SNQ until 2012, data from this region was collected from the PRS from 2006 to 2011.[31] Both registers comprise detailed information on infants treated at neonatal wards. The infants' diagnoses are registered in the SNQ/PRS and MBR according to the International Classification of Diseases, 10th Edition (ICD-10), or as checkboxes in the infant's medical record for some diagnoses in the SNQ. The care at NICUs in Sweden corresponds to the American Academy of Paediatrics' classifications of neonatal care, Levels II to IV.[33] Infants with minor neonatal complications may remain in the maternity ward (equivalent to Level I care) and are not

For peer review only - http://bmjopen.bmj.com/site/about/guidelines.xhtml

included in the SNQ or PRS. Their diagnoses are, however, registered in the MBR. Infant birth weight z-scores were estimated based on infant weight for gestational age and sex according to a Swedish ultrasound-based intrauterine growth curve.[34]

The study was approved by the regional ethical review board in Lund (dnr. 2019-02066). We used the STROBE cohort checklist when writing our report.[35]

Patient and Public Involvement

No patient involved.

Statistical Analyses

Exposures were any antipsychotic use versus no use, use of the different antipsychotic groups versus no use, use of antipsychotics in early and late pregnancy, respectively, versus no use, and use during versus use before or after pregnancy. Outcomes (with ICD-10-codes in parentheses) were admission to NICU (yes/no, duration), transient tachypnoea of the newborn (P221, P228 or checkbox in SNQ), persistent pulmonary hypertension (PPHN) (P293B or checkbox in SNQ), respiratory distress syndrome (P220), hyperbilirubinemia (P58, P59 or checkbox in SNQ), hypoglycaemia (P704 or checkbox in SNQ), feeding difficulties (P92 or checkbox in SNQ), neurological disorders (P909 seizures, P941-42 congenital hyper-/hypotonia, P910-9 disturbances of cerebral status or checkbox in SNQ), withdrawal symptoms (P961-2), any malformations (all Q-codes with exception for Q18, Q65, Q270, Q250 in term infants, or Q53 in term infants), heart malformations (Q20) and need for treatment with continuous positive airway pressure (CPAP) (DG001) or ventilator (DG002). Risk ratios (RRs) for dichotomous outcomes were obtained by using modified Poisson regression in multivariable regression models. Crude and adjusted RRs are displayed. In the final analyses, adjustments were made for: maternal age (continuous), primiparity (versus

For peer review only - http://bmjopen.bmj.com/site/about/guidelines.xhtml

multiparity), maternal smoking (ordinal: 1=no smoking, 2=smoking <10 cigarettes/day, 3=smoking ≥10 cigarettes/day, entered as a continuous variable), BMI (continuous), maternal use of other neurotropic drugs (listed in table 1) and caesarean section (versus vaginal birth). As a sensitivity analysis, the risks were also adjusted for gestational age and Z-score for birth weight to examine how much of the neonatal morbidity could be attributed to these factors (Supplemental Tables 2 and 3). Missing data regarding maternal smoking and BMI were low and replaced by the overall means. (Table 1) For descriptive data, chi-square tests were used to detect heterogeneity between exposure groups. (Table 1)

Length of stay at neonatal ward are presented as medians and interquartile ranges for the three exposure groups. Difference in the length of stay at neonatal ward between exposed and never exposed infants was evaluated by using univariate ANOVA of the logarithmic variable for length of stay that followed the normal distribution. Number needed to harm (NNH) was calculated from the adjusted risk difference between exposed and never exposed infants. For this analysis, the adjusted frequencies of NICU admission, PPHN and neurological disorders amongst the exposed infants were calculated from the results of the multivariate risk analyses for these outcomes, to adjust for the risk of the underlying maternal condition. The statistical analyses were conducted by using SPSS version 27 (IBM SPSS Statistics, IBM Corporation, Armonk, NY).

Results

Among the included infants, 2677 (0.2%) were exposed to antipsychotics during pregnancy, and 34 492 (2.6%) had mothers treated with antipsychotics before or after but not during the pregnancy. The study outline is presented as a flowchart in Figure 1. The most prescribed antipsychotics in the pregnant population during the study period were quetiapine (1021

For peer review only - http://bmjopen.bmj.com/site/about/guidelines.xhtml

exposures), olanzapine (771 exposures) and aripiprazole (334 exposures), all second-generation antipsychotics. The most prescribed first-generation antipsychotic was levomepromazine with 251 exposures. (Supplemental Table 1)

Table 1 summarizes the background characteristics of the included infants and their mothers. The infants exposed to antipsychotics were more likely to be born with a caesarean section and had a significantly higher percentage of mothers who smoked, who were overweight or obese, mothers not living with the father of the child and mothers with concomitant medications such as antidepressants and anxiolytics, compared to both unexposed infants and infants to mothers treated with antipsychotics before and/or after the pregnancy. The exposed infants were also more likely to be small for gestational age. No statistically significant difference between the groups was found in the incidence of preeclampsia, the infant being large for gestational age, perinatal death or infant sex.

The risk of being admitted to neonatal care was increased for infants exposed to antipsychotics in early pregnancy only compared to non-exposed peers, adjusted RR [aRR] 1.5, 95% confidence interval [CI] 1.3-1.7. Exposure in late pregnancy increased the risk further, with aRR 1.8, 95% CI 1.6-2.0. The risk of being admitted to NICU was similar for infants exposed to first- and second-generation antipsychotics (Table 2). When compared to infants born to women treated with antipsychotics before or after but not during pregnancy, the risk increase for admission to NICU was lower but still statistically significant. Additional adjustment for gestational age and Z-score for infant weight did not markedly reduce the risk for being admitted to NICU for any group of infants. (Supplemental Table 2). The NNH for NICU admission compared to the population adjusted for maternal factors was 18.

The neonatal disorders with increased risks associated with antipsychotics exposure were withdrawal symptoms from therapeutic drugs (aRR 17.7, 95% CI 9.6-32.6), neurological

For peer review only - http://bmjopen.bmj.com/site/about/guidelines.xhtml

disorders (aRR 3.4, 95% CI 2.5-4.7) and PPHN (aRR 2.1, 95% CI 1.4-3.1) when compared to the population. (Table 3). However, the absolute frequency of these diagnoses was still less than two percent amongst the exposed infants. A sensitivity analysis on PPHN only including term infants born at gestational week 37 or later did not change the risk estimates. Adjusted NNH for neurological disorders was 135 and for PPHN 458, when compared to the population.

The most frequent outcomes affecting 5-7% of the exposed infants were respiratory disorders, hyperbilirubinemia, and hypoglycaemia. The adjusted risks for these conditions were moderately increased for exposed infants, slightly lower when compared to the control group exposed before/after pregnancy than in comparison to the population. (Table 3) Additional adjustment for fetal factors including prematurity did not markedly change these risks. (Supplemental table 3) No statistically significant differences between the groups were found for respiratory distress syndrome, ventilator treatment, any malformations or heart malformations.

To grade the severity of the neonatal morbidity after exposure to antipsychotics, length of stay was calculated for the full-term infants admitted to NICU. (Table 4) Median length of stay in infants exposed to antipsychotics during pregnancy was 5 days, similar to the infants not exposed. However, when corrected for maternal factors, the exposed infants had slightly but statistically significantly longer length of stay at NICU when compared to infants to mothers never exposed to antipsychotics. For full-term infants with PPHN and/or neurological disorders, the length of stay at NICU was slightly shorter than for the unexposed infants.

Discussion

Neonatal illness and the risk for admission to NICU were found to be increased amongst the exposed infants, at similar levels for both first- and second-generation antipsychotics. The risk

For peer review only

For peer review only - http://bmjopen.bmj.com/site/about/guidelines.xhtml

of needing neonatal care was further increased after exposure in late pregnancy. The equal or slightly longer length of stay at NICU in exposed infants compared to never exposed infants indicate that these admissions reflect real neonatal illness and are not only a cautionary measure due to the exposure. The risk for PPHN was also clearly increased for the exposed infants, which has to our knowledge not been previously described related to antipsychotics.

Our results provide a detailed description of the neonatal morbidity that has not been published before in a study of this size. It is likely that the larger cohort size in this study enables studying the rarer neonatal outcomes including the two overlapping diagnoses of neurological outcomes and drug withdrawal symptoms. These outcomes have not previously been shown to be increased amongst exposed infants, but in this study, they are clearly increased even after adjustments for maternal factors. [22 23] An increase in neonatal morbidity after use of antipsychotics in late pregnancy that we found has also been observed for selective serotonin reuptake inhibitors (SSRIs).[36] Considering the mechanisms of these drugs in the serotonergic and dopaminergic pathways of the central nervous system there is likely to be a biological, drug-related explanation behind this.[37] However, the causality of this connection remains to be clarified.

The Swedish nationwide registers have some limitations regarding data on the drug exposure, that are based on filled prescriptions (PDR) and patient interviews (MBR) and not containing information on to what extent the women took the drugs. It is likely that some degree of exposure misclassification exists. Since information on drug intake was collected before the outcomes were known, it is unlikely the exposure misclassification has seriously biased the results. Even with these draw-backs, we consider the results from this study to be well generalizable to other cohorts, due to the nationwide heterogeneous cohort.

The risk estimates for NICU admission and the studied neonatal disorders decreased but remained significant when exposed infants were compared to infants to mothers treated with

For peer review only - http://bmjopen.bmj.com/site/about/guidelines.xhtml

antipsychotics before or after pregnancy. This indicates that a part of, but not all, the risk-increase for neonatal morbidity seen in the exposed infants is likely to be attributable to underlying factors rather than the drug treatment. However, as the results remained significant after adjustments, it is likely, that there is also a true risk for neonatal morbidity connected to the antipsychotic drug treatment, especially after exposure in late pregnancy. The neonatal disorders seem transient and predominantly mild, and these findings do not warrant discontinuation of a necessary treatment but rather an increased monitoring of these infants postpartum.

For policymakers it is important to consider the needs for a well-developed and structured healthcare for women with psychiatric disorders and their infants. For the clinicians, it is also important to acknowledge the crude risk estimates of this study, as they reflect the true risks for this group of patients, burdened with comorbidities. Whether there is a dose-response relationship to these effects remains to be studied.

The risk-increase for PPHN that was found could be explained by poor adaptation in these infants due to the neurological effects of the drugs, leading to both respiratory disorders and down the line even persistent pulmonary hypertension. Over half of the exposures in our data set consist of olanzapine and quetiapine, two substances affecting serotonergic as well as dopaminergic receptors.[38] A similar, but milder, correlation to PPHN has also been shown for prenatal exposure for SSRIs.[36 39] The correlation did however not sustain after adjustments for prematurity and birth weight, when the exposed infants were compared to infants whose mothers used antipsychotics before or after pregnancy. We speculate that an effect on the serotonergic system might partly explain the increased risk of PPHN. Our conclusion is that the risk seems increased for the exposed infants on group level, but whether it is a causal effect of the antipsychotic drug is yet to be determined, and further studies on this subject are

For peer review only - http://bmjopen.bmj.com/site/about/guidelines.xhtml

needed. Future larger cohorts are also needed to study the neonatal effects of the separate antipsychotic drugs.

Conclusion

Fetal exposure to antipsychotic drugs is associated with an increased risk of admission to NICU and to several neonatal illnesses. The risk for neonatal morbidity seems similar for infants exposed to first- and second- generation antipsychotics, and more pronounced after exposure in late pregnancy. The increased risk for PPHN that was found needs to be studied further.

Author contributions

All authors planned and designed the study together and approved the final manuscript as submitted and agree to be accountable for all aspects of the work.

Prof Källén was responsible for the data collection. Dr Heinonen drafted the initial manuscript. Prof. Källén and drs Forsberg, Nörby and Wide reviewed and revised the manuscript.

Competing interests

The authors have no conflicts of interest relevant to this article to disclose.

Funding

This work was supported by the Swedish Research Council, grant number 521-2012-3466, (EH, KW, LF), Stockholm County Council (ALF) with grant number 573301 (EH, KW, LF). The funders did not participate in the work.

Availability of data and material

Individual data are protected by the General Data Protection Regulation in EU (GDPR). All data is stored and accessible with permission only in a data repository at The Swedish National Board of Health and Welfare.

For peer review only - http://bmjopen.bmj.com/site/about/guidelines.xhtml

Acknowledgements

None

References

1. Alexander GC, Gallagher SA, Mascola A, et al. Increasing off-label use of antipsychotic medications in the United States, 1995-2008. *Pharmacoepidemiology and Drug Safety* 2011;20(2):177-84. doi: 10.1002/pds.2082
2. Stephenson CP, Karanges E, McGregor IS. Trends in the utilisation of psychotropic medications in Australia from 2000 to 2011. *Australian & New Zealand Journal of Psychiatry* 2013;47(1):74-87. doi: 10.1177/0004867412466595
3. Galbally M, Snellen M, Power J. Antipsychotic drugs in pregnancy: a review of their maternal and fetal effects. *Therapeutic Advances in Drug Safety* 2014;5(2):100-09. doi: 10.1177/2042098614522682
4. Hatters Friedman S, Moller-Olsen C, Prakash C, et al. Atypical antipsychotic use and outcomes in an urban maternal mental health service. *The International Journal of Psychiatry in Medicine* 2016;51(6):521-33. doi: 10.1177/0091217417696739
5. Ellfolk M, Leinonen MK, Gissler M, et al. Second-generation antipsychotics and pregnancy complications. *European Journal of Clinical Pharmacology* 2020;76(1):107-15. doi: 10.1007/s00228-019-02769-z
6. Toh S, Li Q, Cheetham TC, et al. Prevalence and trends in the use of antipsychotic medications during pregnancy in the U.S., 2001–2007: a population-based study of 585,615 deliveries. *Archives of Women's Mental Health* 2013;16(2):149-57. doi: 10.1007/s00737-013-0330-6
7. Huybrechts KF, Hernandez-Diaz S, Patorno E, et al. Antipsychotic Use in Pregnancy and the Risk for Congenital Malformations. *JAMA psychiatry* 2016;73(9):938-46. doi: 10.1001/jamapsychiatry.2016.1520
8. Kulkarni J, Storch A, Baraniuk A, et al. Antipsychotic use in pregnancy. *Expert Opinion on Pharmacotherapy* 2015;16(9):1335-45. doi: 10.1517/14656566.2015.1041501
9. Habermann F, Fritzsche J, Fuhlbruck F, et al. Atypical antipsychotic drugs and pregnancy outcome: a prospective, cohort study. *J Clin Psychopharmacol* 2013;33(4):453-62. doi: 10.1097/JCP.0b013e318295fe12 [published Online First: 2013/06/15]
10. McKenna K, Koren G, Tetelbaum M, et al. Pregnancy outcome of women using atypical antipsychotic drugs: a prospective comparative study. *J Clin Psychiatry* 2005;66(4):444-9; quiz 546.
11. Reis M, Kallen B. Maternal use of antipsychotics in early pregnancy and delivery outcome. *J Clin Psychopharmacol* 2008;28(3):279-88. doi: 10.1097/JCP.0b013e318172b8d5 [published Online First: 2008/05/16]
12. Kulkarni J, Worsley R, Gilbert H, et al. A prospective cohort study of antipsychotic medications in pregnancy: the first 147 pregnancies and 100 one year old babies. *PLoS One* 2014;9(5):e94788. doi: 10.1371/journal.pone.0094788
13. Einarson A, Boskovic R. Use and Safety of Antipsychotic Drugs During Pregnancy. *Journal of Psychiatric Practice* 2009;15(3):183-92. doi: 10.1097/01.pra.0000351878.45260.94
14. Gentile S, Fusco ML. Schizophrenia and motherhood. *Psychiatry and Clinical Neurosciences* 2019;73(7):376-85. doi: 10.1111/pcn.12856

For peer review only - http://bmjopen.bmj.com/site/about/guidelines.xhtml

15. Gentile S. Antipsychotic therapy during early and late pregnancy. A systematic review. *Schizophr Bull* 2010;36(3):518-44. doi: 10.1093/schbul/sbn107
16. Kallen B, Borg N, Reis M. The use of central nervous system active drugs during pregnancy. *Pharmaceuticals* 2013;6(10):1221-86. doi: 10.3390/ph6101221
17. Ozdemir AK, Pak SC, Canan F, et al. Paliperidone palmitate use in pregnancy in a woman with schizophrenia. *Arch Womens Ment Health* 2015;18(5):739-40. doi: 10.1007/s00737-014-0496-6
18. Viguera AC, Whitfield T, Baldessarini RJ, et al. Risk of Recurrence in Women With Bipolar Disorder During Pregnancy: Prospective Study of Mood Stabilizer Discontinuation. *American Journal of Psychiatry* 2007;164(12):1817-24. doi: 10.1176/appi.ajp.2007.06101639
19. Newport DJ, Calamaras MR, DeVane CL, et al. Atypical antipsychotic administration during late pregnancy: placental passage and obstetrical outcomes. *Am J Psychiatry* 2007;164(8):1214-20. doi: 10.1176/appi.ajp.2007.06111886
20. Sadowski A, Todorow M, Yazdani Brojeni P, et al. Pregnancy outcomes following maternal exposure to second-generation antipsychotics given with other psychotropic drugs: a cohort study. *BMJ Open* 2013;3(7) doi: 10.1136/bmjopen-2013-003062
21. Terrana N, Koren G, Pivovarov J, et al. Pregnancy Outcomes Following In Utero Exposure to Second-Generation Antipsychotics: A Systematic Review and Meta-Analysis. *J Clin Psychopharmacol* 2015;35(5):559-65. doi: 10.1097/JCP.0000000000000391 [published Online First: 2015/08/15]
22. Vigod SN, Gomes T, Wilton AS, et al. Antipsychotic drug use in pregnancy: high dimensional, propensity matched, population based cohort study. *BMJ* 2015;350:h2298. doi: 10.1136/bmj.h2298
23. Petersen I, McCrea RL, Sammon CJ, et al. Risks and benefits of psychotropic medication in pregnancy: cohort studies based on UK electronic primary care health records. *Health Technol Assess* 2016;20(23):1-176. doi: 10.3310/hta20230 [published Online First: 2016/04/01]
24. Frayne J, Nguyen T, Bennett K, et al. The effects of gestational use of antidepressants and antipsychotics on neonatal outcomes for women with severe mental illness. *Australian and New Zealand Journal of Obstetrics and Gynaecology* 2017;57(5):526-32. doi: 10.1111/ajo.12621
25. Jablensky AV, Morgan V, Zubrick SR, et al. Pregnancy, delivery, and neonatal complications in a population cohort of women with schizophrenia and major affective disorders. *Am J Psychiatry* 2005;162(1):79-91. doi: 10.1176/appi.ajp.162.1.79 [published Online First: 2004/12/31]
26. Boden R, Lundgren M, Brandt L, et al. Risks of adverse pregnancy and birth outcomes in women treated or not treated with mood stabilisers for bipolar disorder: population based cohort study. *BMJ* 2012;345:e7085. doi: 10.1136/bmj.e7085 [published Online First: 2012/11/10]
27. Abel K. HL. Schizophrenia, psychopharamacology and pregnancy. . Galbally M, Snellen M, Lewis A (eds), Psychopharmacology and Pregnancy – Treatment Efficacy, Risks, and Guidelines. Heidelberg: Springer 2014.
28. Epidemiology. Cf. The Swedish Medical Birth Register: a summary of content and quality. [Available from: https://www.socialstyrelsen.se/globalassets/sharepoint-dokument/artikelkatalog/ovrigt/2003-112-3_20031123.pdf accessed November 9 2020.
29. Wettermark B, Hammar N, Michaelfored C, et al. The new Swedish Prescribed Drug Register—Opportunities for pharmacoepidemiological research and experience from the first six months. *Pharmacoepidemiology and Drug Safety* 2007;16(7):726-35. doi: 10.1002/pds.1294
30. Register. SNQ. [accessed November 9 2020.
31. Molin J. A regional perinatal database in southern Sweden--a basis for quality assurance in obstetrics and neonatology. *Acta Obstet Gynecol Scand Suppl* 1997;164:37-39.
32. Ornoy A. Valproic acid in pregnancy: How much are we endangering the embryo and fetus? *Reproductive Toxicology* 2009;28(1):1-10. doi: 10.1016/j.reprotox.2009.02.014

For peer review only - http://bmjopen.bmj.com/site/about/guidelines.xhtml

33. Levels of Neonatal Care. *PEDIATRICS* 2012;130(3):587-97. doi: 10.1542/peds.2012-1999
34. Maršál K, Persson PH, Larsen T, et al. Intrauterine growth curves based on ultrasonically estimated foetal weights. *Acta Paediatrica* 1996;85(7):843-48. doi: 10.1111/j.1651-2227.1996.tb14164.x
35. von Elm E, Altman DG, Egger M, et al. The Strengthening the Reporting of Observational Studies in Epidemiology (STROBE) statement: guidelines for reporting observational studies. *J Clin Epidemiol* 2008;61(4):344-9. doi: 10.1016/j.jclinepi.2007.11.008 [published Online First: 2008/03/04]
36. Norby U, Forsberg L, Wide K, et al. Neonatal Morbidity After Maternal Use of Antidepressant Drugs During Pregnancy. *Pediatrics* 2016;138(5) doi: 10.1542/peds.2016-0181
37. Ritter J. Rang and Dale's pharmacology (Ninth edition.). Edinburgh: Elsevier 2020:592-97.
38. Grinchii D, Dremencov E. Mechanism of Action of Atypical Antipsychotic Drugs in Mood Disorders. *International Journal of Molecular Sciences* 2020;21(24):9532. doi: 10.3390/ijms21249532
39. Kieler H, Artama M, Engeland A, et al. Selective serotonin reuptake inhibitors during pregnancy and risk of persistent pulmonary hypertension in the newborn: population based cohort study from the five Nordic countries. *BMJ* 2012;344:d8012. doi: 10.1136/bmj.d8012 [published Online First: 2012/01/14]

For peer review only

	Anti-psychotics during pregnancy n=2677 %	Antipsychotics before or after but not during pregnancy n=34 492 %	No anti-psychotic use[1] n=1 262 047 %	Antipsychotics during pregnancy vs antipsychotics before/after pregnancy p	Antipsychotics during pregnancy vs no antipsychotic use p	Antipsychotics before/after pregnancy vs no antipsychotic use p
**Year of Child Birth**				<0.001	<0.001	<0.001
2006-2010	37.8	55.9	48.3			
2011-2017	62.2	44.1	51.5			
**Maternal Age, y**				<0.001	0.020	<0.001
<20	2.6	3.1	2			
20-35	70.3	77.2	76.4			
35+	27	19.7	21.6			
**Parity**				<0.001	<0.001	<0.001
Primipara	70.6	60.1	63.2			
Multipara	29.4	39.9	36.2			
**BMI in Early Pregnancy**				<0.001	<0.001	<0.001
<24.9	39.8	50.1	56.8			
25-29.9	28.8	24.7	23.6			
>=30	23.1	16.6	11.7			
BMI Unknown	8.3	8.6	7.8			
**Smoking in Early Pregnancy**				<0.001	<0.001	<0.001
No	67.9	77.6	89.4			
Yes	27.4	17.2	5.3			
Missing information	4.7	5.2	5.3			
**Maternal Country of Birth**				<0.001	0.209	<0.001
Sweden	75.4	80.2	73.6			
Other nordic	4.6	2.7	4.1			
Non-nordic	19.6	16.9	20.4			
**Maternal Cohabitation**				<0.001	<0.001	<0.001
Not living with father of child	24.5	14	6			
**Concomitant Neurotropic Drugs**						
Lithium (N05A N01)	4.6	0.8	0	<0.001	<0.001	<0.001
Opioids (N02A)	1.6	1.8	0.4	0.680	<0.001	<0.001
Antiepileptics (N03A)	8	2.1	0.2	<0.001	<0.001	<0.001
Antidepressants (N06A)	28.2	12.8	1.9	<0.001	<0.001	<0.001
Psychostimulants (N06B)	3.3	1	0.1	<0.001	<0.001	<0.001
Anxiolytics and sedatives (N05B, C)	16.3	4.1	0.4	<0.001	<0.001	<0.001
Milder anxiolytics and antiemetics[2]	13.7	7.3	1	<0.001	<0.001	<0.001
**Pregnancy Complications**						
Gestational Diabetes	2.8	1.4	1.2	<0.001	<0.001	<0.001
Preeclampsia	0.9	1	0.8	0.729	0.512	<0.001
Cesarean Section	25.7	20.8	16.4	<0.001	<0.001	<0.001
Prematurity	9.7	7.2	5.3	<0.001	<0.001	<0.001
Small for Gestational Age	3.9	2.7	2.3	<0.001	<0.001	<0.001
Large for Gestational Age	4.7	4.1	4	0.121	0.060	0.386
APGAR 5min <7	3.4	2.1	1.4	<0.001	<0.001	<0.001
Perinatal Death	0.6	0.5	0.4	0.357	0.108	0.046
**Infant Sex**				0.777	0.610	0.454
Male	51.0	51.2	51.1			
Female	49.0	48.8	48.9			

Table 1. Background Characteristics of the Study Population. [1] No use during the entire study period. [2] Including Promethazine (R06AD02, R06AD52), Alimemazine (R06AD01), Dixyrazine (N05A B01), Prochlorperazine (N05A B04) and Melperone (N05A D03)

For peer review only

	NICU Admissions	Exposed vs Never exposed				Exposed Infants vs Infants to Mothers Treated with Antipsychotics Before or After the Pregnancy			
	n (%)	Crude RR	95% CI	Adj. RR	95% CI	Crude RR	95% CI	Adj. RR	95% CI
**Exposed early pregnancy only**									
Any Antipsychotics, n=1454	251 (17.3)	2.2	2.0-2.5	1.5	1.3-1.7	1.5	1.3-1.7	1.3	1.1-1.4
First-generation, n=431	76 (17.6)	2.2	1.7-2.7	1.5	1.2-1.9	1.5	1.2-1.8	1.3	1.0-1.6
Second-generation, n=1166	212 (18.2)	2.2	2.0-2.6	1.5	1.3-1.7	1.5	1.3-1.7	1.3	1.1-1.4
**Exposed in late pregnancy**									
Any Antipsychotics, n=1223	265 (21.7)	2.8	2.5-3.1	1.8	1.6-2.0	1.9	1.7-2.1	1.5	1.4-1.7
First-generation, n=297	73 (24.6)	3.1	2.6-3.8	2.1	1.7-2.5	2.1	1.7-2.6	1.7	1.4-2.1
Second-generation, n=972	208(21.4)	2.7	2.4-3.1	1.8	1.6-2.0	1.9	1.6-2.1	1.5	1.3-1.7
**Never exposed, n=1262 047**	98976 (7.8)	REFERENCE GROUP 1							
**Exposed before or after but not during pregnancy, n=34 492**	4010 (11.6)	REFERENCE GROUP 2							

Table 2. Risk ratio (RR) for admission to neonatal intensive care unit (NICU) compared with non-exposed infants and with infants to mothers using antipsychotics before and/or after but not during the current pregnancy. Adjusted for: Primipara, Age, Body Mass Index (BMI), Smoking, Cesarean section, Concurrent neurotropic drugs.

For peer review only - http://bmjopen.bmj.com/site/about/guidelines.xhtml

Infant outcomes	Exposed during pregnancy N=2677	Exposed before/after but not during pregnancy N=34 492	Never exposed N=1 262 047	Exposed vs Never exposed				Exposed Infants vs Infants to Mothers Treated with Antipsychotics Before or After the Pregnancy			
	n (%)	n (%)	n (%)	Crude RR	95% CI	Adj. RR	95% CI	Crude RR	95% CI	Adj. RR	95% CI
**Any birth defect** [a]	64/2378 (2.7)	27122 (2.1)	822 (2.4)	1.3	1.0-1.6	1.1	0.8-1.4	1.1	0.9-1.5	1.1	0.8-1.4
Any heart defect [a]	23/2378 (1.0)	9644 (0.8)	304 (0.9)	1.3	0.8-1.9	1.1	0.7-1.6	1.1	0.7-1.7	1.0	0.7-1.6
**Admission to NICU**	516 (19.3)	4010 (11.6)	98976 (7.8)	2.5	2.3-2.7	1.7	1.6-1.8	1.7	1.5-1.8	1.4	1.3-1.5
**Respiratory symptoms**											
Transient tachypnea of the newborn	185 (6.9)	1406 (4.1)	34204 (2.7)	2.5	2.2-2.9	1.6	1.4-1.9	1.7	1.5-2.0	1.4	1.2-1.7
Persistent pulmonary hypertension	27 (1.0)	209 (0.6)	4581 (0.4)	2.8	1.9-4.1	2.1	1.4-3.1	1.7	1.1-2.5	1.5	1.0-2.2
Respiratory distress syndrome	21 (0.8)	296 (0.9)	6671 (0.5)	1.5	1.0-2.3	1.0	0.7-1.6	0.9	0.6-1.4	0.9	0.6-1.4
**Respiratory treatment**											
CPAP	168 (6.3)	1276 (3.7)	29977 (2.4)	2.7	2.3-3.1	1.7	1.4-2.0	1.7	1.5-2.0	1.5	1.2-1.7
Ventilator treatment	22 (0.8)	299 (0.9)	6811 (0.5)	1.5	1.0-2.3	1.1	0.7-1.6	1.0	0.6-1.5	0.9	0.6-1.4
**Hyperbilirubinemia**	169 (6.3)	1819 (5.3)	54563 (4.3)	1.5	1.3-1.7	1.3	1.1-1.5	1.2	1.0-1.4[b]	1.1	1.0-1.3
**Hypoglycemia**	133 (5.0)	1090 (3.2)	28117 (2.2)	2.2	1.9-2.6	1.4	1.2-1.7	1.6	1.3-1.9	1.3	1.1-1.5
**Feeding difficulties**	75 (2.8)	558 (1.6)	14147 (1.1)	2.5	2.0-3.1	1.8	1.4-2.3	1.7	1.4-2.2	1.6	1.2-2.0
**Neurological disorders**	47 (1.8)	186 (0.5)	3821 (0.3)	5.8	4.4-7.7	3.4	2.5-4.7	3.3	2.4-4.5	2.3	1.6-3.3
**Withdrawal symptoms from therapeutic drugs**	34 (1.3)	82 (0.2)	153 (0.0)	105	72.4-152	17.7	9.6-32.6	5.3	3.6-8.0	3.3	2.1-5.3

For peer review only - http://bmjopen.bmj.com/site/about/guidelines.xhtml

Table 3. Risk ratio (RR) for birth defects and neonatal morbidity after exposure to antipsychotics. NICU = Neonatal intensive Care Unit. CPAP = Continuous positive airway pressure. Adjusted for: Primipara, Age, Body Mass Index (BMI), Smoking, Cesarean section, Concurrent neurotropic drugs. [a] Exposures are only counted if present in early pregnancy (n=2378). [b] $p < 0.05$

Length of stay amongst term infants admitted to NICU (days)	All term infants		Infants with diagnosis of PPHN		Infants with neurological disorders	
	n	Median (IQ-range)	n	Median (IQ-range)	n	Median (IQ-range)
Exposed	336	5 (3-9)	12	8 (3-9)	34	9 (6-11)
Exposed before/after pregnancy	2380	5 (2-8)	80	9 (6-15)	129	11 (7-18)
Not exposed	59 439	5 (2-8)	1894	9 (5-17)	2698	11 (7-18)

Table 4. Length of Stay at Neonatal Intensive Care Unit (NICU) amongst term infants presented in median days and interquartile (IQ)-ranges. Presented for all term infants, infants with persistent pulmonary hypertension (PPHN) and infants with neurological disorder.

Figure 1. Flowchart over the study design. 1) Women with pre-pregnancy Diabetes mellitus. 2) Use of Valproate during pregnancy. NICU = Neonatal Intensive Care Unit.

For peer review only - http://bmjopen.bmj.com/site/about/guidelines.xhtml

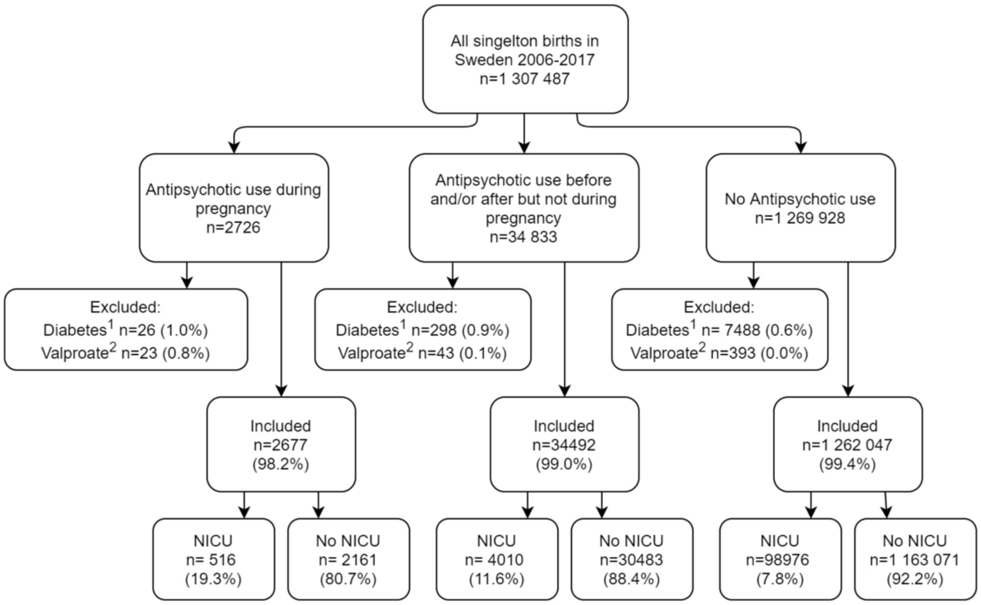

Figure 1. Flowchart over the study design. 1) Women with pre-pregnancy Diabetes mellitus. 2) Use of Valproate during pregnancy. NICU = Neonatal Intensive Care Unit.

522x325mm (300 x 300 DPI)

Supplemental material for: Heinonen et al. Neonatal morbidity after fetal exposure to antipsychotics - a national register-based study

EXPOSURE	n
**First-generation antipsychotics**	
Levomepromazine	251
Flupentixol	165
Haloperidol	162
Perphenazine	93
Zuclopenthixol	75
Chlorprothixene	45
Chlorpromazine	4
Pimozide	4
Fluphenazine	3
Thioridazine	1
**Second-generation antipsychotics**	
Quetiapine	1026
Olanzapine	771
Aripiprazole	334
Risperidone	191
Ziprasidone	34
Clozapine	29
Paliperidone	9
Sertrindole	2
**TOTAL**	3199

Supplemental Table 1. Prescriptions of antipsychotics during pregnancy 2006-2017.

For peer review only

NICU-admissions	Exposed vs Never exposed						Exposed Infants vs Infants to Mothers Treated with Antipsychotics Before or After the Pregnancy					
	Crude		Adjusted for Maternal Factors Only		Adjusted for Maternal and Fetal Factors		Crude		Adjusted for Maternal Factors Only		Adjusted for Maternal and Fetal Factors	
	RR	95% CI	RR	95% CI	RR	95% CI	RR	95% CI	RR	95% CI	RR	95% CI
**Exposed early pregnancy only**												
Any Antipsychotics, n=1454	2.2	2.0-2.5	1.5	1.3-1.7	1.3	1.1-1.6	1.5	1.3-1.7	1.3	1.1-1.4	1.2	1.0-1.4
F-GA, n=431	2.2	1.7-2.7	1.5	1.2-1.9	1.5	1.2-1.8	1.5	1.2-1.8	1.3	1.0-1.6	1.3	1.0-1.6*
S-GA, n=1166	2.3	2.0-2.6	1.5	1.3-1.7	1.3	1.0-1.7*	1.5	1.3-1.7	1.3	1.1-1.4	1.2	0.9-1.4
**Exposed in late pregnancy**												
Any Antipsychotics, n=1223	2.8	2.5-3.1	1.8	1.6-2.0	1.8	1.6-2.0	1.9	1.7-2.1	1.5	1.4-1.7	1.5	1.4-1.7
F-GA, n=297	3.1	2.6-3.8	2.1	1.7-2.5	2.1	1.7-2.5	2.1	1.7-2.6	1.7	1.4-2.1	1.7	1.4-2.0
S-GA, n=972	2.7	2.4-3.1	1.8	1.6-2.0	1.8	1.6-2.0	1.9	1.6-2.1	1.5	1.3-1.7	1.5	1.3-1.7
**Never exposed, n=1262 047**	REFERENCE GROUP 1											
**Exposed before or after but not during pregnancy, n=34 492**	REFERENCE GROUP 2											

Supplemental table 2. Risk (RR) for admission to neonatal intensive care unit (NICU) compared with non-exposed infants and with infants to mothers using antipsychotics before and/or after but not during the current pregnancy. F-GA = First-generation Antipsychotics, S-GA = Second-generation Antipsychotics. Maternal factors: Primipara, Age, Body Mass Index (BMI), Smoking, Cesarean section, Concurrent neurotropic drugs. Fetal factors: Gestational Age, Z-score. * p <0.05

For peer review only

Neonatal outcomes	Exposed vs Never exposed						Exposed Infants vs Infants to Mothers Treated with Antipsychotics Before or After the Pregnancy					
	Crude		Adjusted for Maternal Factors Only		Adjusted for Maternal and Fetal Factors		Crude		Adjusted for Maternal Factors Only		Adjusted for Maternal and Fetal Factors	
	RR	95% CI	RR	95% CI	RR	95% CI	RR	95% CI	RR	95% CI	RR	95% CI
**Admission to NICU**	2.5	2.3-2.7	1.7	1.6-1.8	1.6	1.4-1.6	1.7	1.5-1.8	1.4	1.3-1.5	1.4	1.2-1.5
**Respiratory symptoms**												
Transient tachypnea of the newborn	2.5	2.2-2.9	1.6	1.4-1.9	1.5	1.3-1.8	1.7	1.5-2.0	1.4	1.2-1.7	1.4	1.2-1.6
Persistent pulmonary hypertension	2.8	1.9-4.1	2.1	1.4-3.1	1.5	0.9-2.6	1.7	1.1-2.5	1.5	1.0-2.2	1.2	0.7-2.2
Respiratory distress syndrome	1.5	1.0-2.3	1.0	0.7-1.6	0.7	0.3-1.5	0.9	0.6-1.4	0.9	0.6-1.4	0.6	0.3-1.3
**Respiratory treatment**												
CPAP	2.7	2.3-3.1	1.7	1.4-2.0	1.5	1.1-2.0	1.7	1.5-2.0	1.5	1.2-1.7	1.3	1.0-1.7
Ventilator treatment	1.5	1.0-2.3	1.1	0.7-1.6	0.8	0.4-1.5	1.0	0.6-1.5	0.9	0.6-1.4	0.7	0.3-1.4
**Hyperbilirubinemia**	1.5	1.3-1.7	1.3	1.1-1.5	1.2	0.9-1.4	1.2	1.0-1.4*	1.1	1.0-1.3	1.1	0.9-1.3
**Hypoglycemia**	2.2	1.9-2.6	1.4	1.2-1.7	1.3	1.1-1.6	1.6	1.3-1.9	1.3	1.1-1.5	1.2	1.0-1.5*
**Feeding difficulties**	2.5	2.0-3.1	1.8	1.4-2.3	1.7	1.3-2.2	1.7	1.4-2.2	1.6	1.2-2.0	1.5	1.1-1.9
**Neurological disorders**	5.8	4.4-7.7	3.4	2.5-4.7	3.3	2.4-4.6	3.3	2.4-4.5	2.3	1.6-3.3	2.3	1.6-3.3
**Withdrawal symptoms from therapeutic drugs**	105	72.4-152	17.7	9.6-32.6	17.3	9.4-31.7	5.3	3.6-8.0	3.3	2.1-5.3	3.4	2.1-5.3

Supplemental table 3. Risk (RR) for neonatal morbidity after exposure to antipsychotics. NICU = Neonatal intensive Care Unit. CPAP = Continuous positive airway pressure. Maternal factors: Primipara, Age, Body Mass Index (BMI), Smoking, Cesarean section, Concurrent neurotropic drugs. Fetal factors: Gestational Age, Z-score. * p <0.05

For peer review only

Reporting checklist for cohort study.

Based on the STROBE cohort guidelines.

Instructions to authors

Complete this checklist by entering the page numbers from your manuscript where readers will find each of the items listed below.

Your article may not currently address all the items on the checklist. Please modify your text to include the missing information. If you are certain that an item does not apply, please write "n/a" and provide a short explanation.

Upload your completed checklist as an extra file when you submit to a journal.

In your methods section, say that you used the STROBE cohortreporting guidelines, and cite them as:

von Elm E, Altman DG, Egger M, Pocock SJ, Gotzsche PC, Vandenbroucke JP. The Strengthening the Reporting of Observational Studies in Epidemiology (STROBE) Statement: guidelines for reporting observational studies.

		Reporting Item	Page Number
**Title and abstract**			
Title	#1a	Indicate the study's design with a commonly used term in the title or the abstract	1
Abstract	#1b	Provide in the abstract an informative and balanced summary of what was done and what was found	2
**Introduction**			
Background / rationale	#2	Explain the scientific background and rationale for the investigation being reported	3
Objectives	#3	State specific objectives, including any prespecified hypotheses	3
**Methods**			
Study design	#4	Present key elements of study design early in the paper	4
Setting	#5	Describe the setting, locations, and relevant dates, including periods of recruitment, exposure, follow-up, and data collection	4

For peer review only

Eligibility criteria	#6a	Give the eligibility criteria, and the sources and methods of selection of participants. Describe methods of follow-up.	4
Eligibility criteria	#6b	For matched studies, give matching criteria and number of exposed and unexposed	4
Variables	#7	Clearly define all outcomes, exposures, predictors, potential confounders, and effect modifiers. Give diagnostic criteria, if applicable	6
Data sources / measurement	#8	For each variable of interest give sources of data and details of methods of assessment (measurement). Describe comparability of assessment methods if there is more than one group. Give information separately for for exposed and unexposed groups if applicable.	66
Bias	#9	Describe any efforts to address potential sources of bias	6
Study size	#10	Explain how the study size was arrived at	4
Quantitative variables	#11	Explain how quantitative variables were handled in the analyses. If applicable, describe which groupings were chosen, and why	6
Statistical methods	#12a	Describe all statistical methods, including those used to control for confounding	
6,7			
Statistical methods	#12b	Describe any methods used to examine subgroups and interactions	7
Statistical methods	#12c	Explain how missing data were addressed	7
Statistical methods	#12d	If applicable, explain how loss to follow-up was addressed	NA
Statistical methods	#12e	Describe any sensitivity analyses	
7			
**Results**			
Participants	#13a	Report numbers of individuals at each stage of study—eg numbers potentially eligible, examined for eligibility, confirmed eligible,	Figure 1

For peer review only

		included in the study, completing follow-up, and analysed. Give information separately for for exposed and unexposed groups if applicable.	
Participants	#13b	Give reasons for non-participation at each stage	Figure 1
Participants	#13c	Consider use of a flow diagram	
Figure 1			
Descriptive data	#14a	Give characteristics of study participants (eg demographic, clinical, social) and information on exposures and potential confounders. Give information separately for exposed and unexposed groups if applicable.	Table 1
Descriptive data	#14b	Indicate number of participants with missing data for each variable of interest	
16			
Descriptive data	#14c	Summarise follow-up time (eg, average and total amount)	
5			
Outcome data	#15	Report numbers of outcome events or summary measures over time. Give information separately for exposed and unexposed groups if applicable.	
17-19			
Main results	#16a	Give unadjusted estimates and, if applicable, confounder-adjusted estimates and their precision (eg, 95% confidence interval). Make clear which confounders were adjusted for and why they were included	8, 9, 17-19
Main results	#16b	Report category boundaries when continuous variables were categorized	6
Main results	#16c	If relevant, consider translating estimates of relative risk into absolute risk for a meaningful time period	
17-18			
Other analyses	#17	Report other analyses done—eg analyses of subgroups and interactions, and sensitivity analyses	8,9, Supplemental figures 2 & 3

For peer review only

Discussion

Key results	#18	Summarise key results with reference to study objectives	9
Limitations	#19	Discuss limitations of the study, taking into account sources of potential bias or imprecision. Discuss both direction and magnitude of any potential bias.	10
Interpretation	#20	Give a cautious overall interpretation considering objectives, limitations, multiplicity of analyses, results from similar studies, and other relevant evidence.	11
Generalisability	#21	Discuss the generalisability (external validity) of the study results	10

Other Information

Funding	#22	Give the source of funding and the role of the funders for the present study and, if applicable, for the original study on which the present article is based	12

Notes:

- 16a: 8, 9, 17-19
- 17: 8,9, Supplemental figures 2 & 3 The STROBE checklist is distributed under the terms of the Creative Commons Attribution License CC-BY. This checklist was completed on 21. January 2022 using https://www.goodreports.org/, a tool made by the EQUATOR Network in collaboration with Penelope.ai

For peer review only

BMJ Open

Neonatal morbidity after fetal exposure to antipsychotics - a national register-based study

Journal:	*BMJ Open*
Manuscript ID	bmjopen-2022-061328.R1
Article Type:	Original research
Date Submitted by the Author:	03-May-2022
Complete List of Authors:	Heinonen, Essi; Karolinska institutet Department of Clinical Sciences Intervention and Technology, Pediatrics; Karolinska University Hospital, Department of Neonatology<br>Forsberg, Lisa; Karolinska institutet Department of Clinical Sciences Intervention and Technology, Pediatrics<br>Nörby, Ulrika ; Lund University, Department of Clinical Sciences, Centre of Reproduction Epidemiology, Tornblad Institute; Region Stockholm, Health and Medical Care Administration<br>Wide, Katarina; Karolinska Institute Department of Clinical Science Intervention and Technology, Paediatrics; Karolinska Universitetssjukhuset, Paediatrics<br>Källén, Karin; Lunds Universitet, Department of Clinical Sciences, Centre of Reproduction Epidemiology, Tornblad Institute
**Primary Subject Heading**:	Epidemiology
Secondary Subject Heading:	Obstetrics and gynaecology, Pharmacology and therapeutics, Paediatrics, Mental health
Keywords:	CLINICAL PHARMACOLOGY, EPIDEMIOLOGY, NEONATOLOGY, Maternal medicine < OBSTETRICS, Schizophrenia & psychotic disorders < PSYCHIATRY, Depression & mood disorders < PSYCHIATRY

SCHOLARONE™
Manuscripts

I, the Submitting Author has the right to grant and does grant on behalf of all authors of the Work (as defined in the below author licence), an exclusive licence and/or a non-exclusive licence for contributions from authors who are: i) UK Crown employees; ii) where BMJ has agreed a CC-BY licence shall apply, and/or iii) in accordance with the terms applicable for US Federal Government officers or employees acting as part of their official duties; on a worldwide, perpetual, irrevocable, royalty-free basis to BMJ Publishing Group Ltd ("BMJ") its licensees and where the relevant Journal is co-owned by BMJ to the co-owners of the Journal, to publish the Work in this journal and any other BMJ products and to exploit all rights, as set out in our licence.

The Submitting Author accepts and understands that any supply made under these terms is made by BMJ to the Submitting Author unless you are acting as an employee on behalf of your employer or a postgraduate student of an affiliated institution which is paying any applicable article publishing charge ("APC") for Open Access articles. Where the Submitting Author wishes to make the Work available on an Open Access basis (and intends to pay the relevant APC), the terms of reuse of such Open Access shall be governed by a Creative Commons licence – details of these licences and which Creative Commons licence will apply to this Work are set out in our licence referred to above.

Other than as permitted in any relevant BMJ Author's Self Archiving Policies, I confirm this Work has not been accepted for publication elsewhere, is not being considered for publication elsewhere and does not duplicate material already published. I confirm all authors consent to publication of this Work and authorise the granting of this licence.

For peer review only

Neonatal morbidity after fetal exposure to antipsychotics - a national register-based study

Essi Heinonen, MD,[a,b] **Lisa Forsberg, MD, PhD,**[a] ***Ulrika Nörby,*** **MSc Pharm, PhD,**[d,e] ***Katarina Wide, MD, PhD,***[a,c] ***Karin Källén, Prof.***[d]

[a] Division of Pediatrics, Department of Clinical Science, Intervention and Technology (CLINTEC), Karolinska Institutet, Stockholm, Sweden; [b] *Department of Pediatrics and Newborn medicine at Karolinska University Hospital, Stockholm, Sweden;* [c] *Department of Pediatrics and Emergency Pediatrics at Karolinska University Hospital, Stockholm, Sweden;* [d] Centre of Reproduction Epidemiology, Tornblad Institute, Department of Clinical Sciences, Lund University, Lund, Sweden; [e] Health and Medical Care Administration, Region Stockholm, Stockholm, Sweden.

Address correspondence to: Essi Heinonen, Division of Pediatrics, Department of Clinical Science, Intervention and Technology (CLINTEC), Karolinska Institutet, Stockholm, Sweden. Blickagången 6A, 14157 Huddinge, Sweden. [essi.heinonen@ki.se], +4673-563 3330.

For peer review only

For peer review only - http://bmjopen.bmj.com/site/about/guidelines.xhtml

Abstract

Objective: To investigate the admission rate to neonatal care and neonatal morbidity after maternal use of antipsychotics during pregnancy.

Design: A population-based register study.

Setting: Information on all singleton births between July 2006 and December 2017 in Sweden including data on prescription drugs, deliveries and infants' health were obtained from the Swedish Medical Birth Register, the Prescribed Drug Register, and the Swedish Neonatal Quality Register. Exposed infants were compared to unexposed infants and to infants to mothers treated with antipsychotics before or after but not during pregnancy.

Participants: The cohort comprised a total of 1 307 487 infants, of whom 2677 (0.2%) were exposed to antipsychotics during pregnancy and 34 492 (2.6%) had mothers that were treated before/after the pregnancy.

Outcome measures: The primary outcome was admission rate to neonatal care. Secondary outcomes were the separate neonatal morbidities.

Results: Of the exposed infants, 516 (19.3%) were admitted to neonatal care compared with 98 976 (7.8%) of the unexposed infants (adjusted risk ratio [aRR]: 1.7; 95% confidence interval [CI]: 1.6-1.8), with a further increased risk after exposure in late pregnancy. The highest relative risks were seen for withdrawal symptoms (aRR: 17.7; 95% CI: 9.6-32.6], neurological disorders (aRR: 3.4; 95% CI: 2.4-5.7) and persistent pulmonary hypertension, aRR: 2.1; 95% CI:1.4-3.1) when compared to unexposed infants. The absolute risks for these outcomes were however low amongst the exposed infants, 1.3, 1.8 and 1.0%, respectively, and the relative risks were lower when compared to infants to mothers treated before/after the pregnancy.

Conclusion: Fetal exposure to antipsychotics was associated with an increased risk for neonatal morbidity. The effects in the exposed infants seem transient and predominantly mild, and these findings do not warrant discontinuation of a necessary treatment but rather increased monitoring of these infants. The increased risk for persistent pulmonary hypertension requires further studies.

Article summary

Strengths and limitations of this study

- To our knowledge, this is the largest study to date to investigate the risk for individual neonatal morbidities amongst infants exposed to antipsychotics.
- The Swedish health registers have the advantage of almost complete coverage of the inhabitants, information on important confounders and minimal bias in data collection, with the Swedish Neonatal Quality Register containing detailed information on all infants admitted to neonatal care.
- We included a control group of infants to mothers treated with antipsychotics before or after but not during pregnancy that allowed us to adjust for factors associated to the underlying disorder, at least to some extent.

For peer review only - http://bmjopen.bmj.com/site/about/guidelines.xhtml

- Even though the study included 1.3 million births, the numbers were too low to obtain reliable risk estimates for specific antipsychotics. Instead, the analyses were limited to antipsychotics on group level.

For peer review only

For peer review only - http://bmjopen.bmj.com/site/about/guidelines.xhtml

Introduction

Use of antipsychotic drugs is increasing globally due to the widened indications and off-label use of second generation antipsychotics in treatment of a wide range of psychiatric disorders, also during pregnancy.[1-6] Treatment during pregnancy does not generally seem to increase the risk for malformations.[7-12] Some studies have indicated, that schizophrenia itself might be connected to an increased risk for malformations, and discontinuation of drug therapy is not recommended during pregnancy due to the risk of relapse of the underlying condition. [13-18]

Fetal exposure to antipsychotics has in smaller cohorts been linked to increased risks for prematurity, altered fetal growth, increased admission to neonatal intensive care unit (NICU), low APGAR-scores, and neonatal extrapyramidal and respiratory symptoms. These risks seem to increase with increased dosage and with polypharmacy. Several of the previous studies have not adjusted the risks for any confounding maternal factors.[9 12 16 19-21] Three studies, that adjusted for health- and lifestyle confounders in the analyses, did not confirm the increased neonatal morbidity after intrauterine antipsychotic exposure. Instead, one of them found a correlation between maternal psychiatric care during pregnancy and infant admission to NICU.[22-24] Maternal schizophrenia and bipolar disorders have independent of drug exposure been associated with increased risks of prematurity, preeclampsia, neonatal morbidity including poor arousal and potential adverse neurodevelopmental effects in the infants.[3 14 25-27] Mothers with untreated psychiatric illness are also more likely to engage in high-risk behaviours such as smoking, alcohol and illicit drugs and are less likely to attend to antenatal care.[25]

The aim of this Swedish population based register study was to clarify the pattern and the frequency of the neonatal effects associated with intrauterine antipsychotic exposure. We also attempted to separate these effects from the impact of the underlying disease, its associated risk factors and other medications.

For peer review only - http://bmjopen.bmj.com/site/about/guidelines.xhtml

Methods

The study was a register-based study combining data from the Swedish Medical Birth Register (MBR),[28] the Prescribed Drug Register (PDR),[29] The Swedish Neonatal Quality Register (SNQ),[30] and the Perinatal Revision South Register (PRS).[31] Swedish personal identification numbers were used for register linkages. The study population consisted of all singleton births in Sweden, a total of 1 307 487 infants, registered in the MBR between July 1, 2006, And December 31, 2017. An outline of the study design is presented in Figure 1.

The MBR holds data on antenatal care, delivery, and examination of the newborn for >97% of all births. Information on body mass index (BMI), medications and smoking habits are registered in the MBR at the first visit of the complimentary antenatal care offered all pregnant women in Sweden.[28] For this study, information on drug exposure and maternal and fetal background characteristics were collected from the MBR. Women with a diagnosis of pre-pregnancy diabetes or with valproate treatment (N03A G01) were excluded from the analysis. Valproate exposed infants were excluded due to the well-known teratogenicity of the drug as well as co-medication with valproate and antipsychotics being fairly common.[32]

Data on exposure for prescription drugs were also acquired from the PDR. The PDR stores data on all drugs prescribed in ambulatory care and dispensed at a Swedish pharmacy but does not include medications used in hospitals.[29] The drugs registered in the MBR and PDR are classified according to the Anatomical Therapeutic Chemical (ATC) classification system. Antipsychotic exposure was defined as drugs belonging to ATC-class N05A, antipsychotics. Exposures to the antipsychotics dixyrazine (N05AB01), prochlorperazine (N05AB04), melperone (N05AD03) and lithium (N05A N01) were excluded from the exposed group and considered as covariates, due to the use of these drugs as antiemetics (dixyrazine,

For peer review only - http://bmjopen.bmj.com/site/about/guidelines.xhtml

prochlorperazine, melperone) and as a mood stabilizer (lithium) rather than antipsychotics. The remaining drugs in class N05A were divided into first- and second-generation antipsychotics according to Supplemental Table 1.

Antipsychotics exposure was allocated into any exposure (drugs dispensed at any time during or 1 month before the pregnancy), late exposure (drugs dispensed during the last 90 days of the pregnancy with or without earlier dispenses), and early exposure only (drugs dispensed 1 month before and during pregnancy but not during the last 90 days of the pregnancy). We also created a reference group with women exposed to antipsychotics any time during the study period, before or after the pregnancy, but not during the pregnancy or 1 month before it. This group was used to attempt to control for the effect of the underlying psychiatric illness and other psychosocial factors connected to the neonatal outcome. Exposure data was also collected on the following neurotropic drugs known to or suspected to cause similar neonatal morbidity as antipsychotics: antidepressants (ATC-code N06A), antiepileptics (N03A), opioids (N02A), centrally acting sympathomimetics (N06BA), sedatives (N05B, N05C) and milder sedatives (alimemazine, promethazine and the excluded antiemetic antipsychotics from N05A). Concomitant neurotropic drug exposure was defined as a prescription of these drugs dispensed at any time during or 1 month before the pregnancy and was adjusted for in the multivariable regression model.

Data on NICU admissions and neonatal outcomes were extracted from the SNQ that covers all 37 Swedish NICUs. [30] Because the south of Sweden was not included in the SNQ until 2012, data from this region was collected from the PRS from 2006 to 2011.[31] Both registers comprise detailed information on infants treated at neonatal wards. The infants' diagnoses are registered in the SNQ/PRS and MBR according to the International Classification of Diseases, 10th Edition (ICD-10), or as checkboxes in the infant's medical record for some diagnoses in the SNQ. The care at NICUs in Sweden corresponds to the American Academy of

For peer review only - http://bmjopen.bmj.com/site/about/guidelines.xhtml

Paediatrics' classifications of neonatal care, Levels II to IV.[33] Infants with minor neonatal complications may remain in the maternity ward (equivalent to Level I care) and are not included in the SNQ or PRS. Their diagnoses are, however, registered in the MBR. Infant birth weight z-scores were estimated based on infant weight for gestational age and sex according to a Swedish ultrasound-based intrauterine growth curve.[34]

The study was approved by the regional ethical review board in Lund (dnr. 2019-02066). We used the STROBE cohort checklist when writing our report.[35]

Patient and Public Involvement

No patient involved.

Statistical Analyses

Exposures were any antipsychotic use versus no use, use of the different antipsychotic groups versus no use, use of antipsychotics in early and late pregnancy, respectively, versus no use, and use during versus use before or after pregnancy. Outcomes (with ICD-10-codes in parentheses) were admission to NICU (yes/no, duration), transient tachypnoea of the newborn (P221, P228 or checkbox in SNQ), persistent pulmonary hypertension (PPHN) (P293B or checkbox in SNQ), respiratory distress syndrome (P220), hyperbilirubinemia (P58, P59 or checkbox in SNQ), hypoglycaemia (P704 or checkbox in SNQ), feeding difficulties (P92 or checkbox in SNQ), neurological disorders (P909 seizures, P941-42 congenital hyper- /hypotonia, P910-9 disturbances of cerebral status or checkbox in SNQ), withdrawal symptoms (P961-2), any malformations (all Q-codes with exception for Q18, Q65, Q270, Q250 in term infants, or Q53 in term infants), heart malformations (Q20) and need for treatment with continuous positive airway pressure (CPAP) (DG001) or ventilator (DG002). Risk ratios (RRs) for dichotomous outcomes were obtained by using modified Poisson

For peer review only - http://bmjopen.bmj.com/site/about/guidelines.xhtml

regression in multivariable regression models. Crude and adjusted RRs are displayed. In the final analyses, adjustments were made for: maternal age (continuous), primiparity (versus multiparity), maternal smoking (ordinal: 1=no smoking, 2=smoking <10 cigarettes/day, 3=smoking ≥10 cigarettes/day, entered as a continuous variable), BMI (continuous), maternal use of other neurotropic drugs (listed in table 1) and caesarean section (versus vaginal birth). As a sensitivity analysis, the risks were also adjusted for gestational age and Z-score for birth weight to examine how much of the neonatal morbidity could be attributed to these factors (Supplemental Tables 2 and 3). Missing data regarding maternal smoking and BMI were low and replaced by the overall means. (Table 1) For descriptive data, chi-square tests were used to detect heterogeneity between exposure groups. (Table 1)

Length of stay at neonatal ward are presented as medians and interquartile ranges for the three exposure groups. Difference in the length of stay at neonatal ward between exposed and never exposed infants was evaluated by using univariate ANOVA of the logarithmic variable for length of stay that followed the normal distribution. Number needed to harm (NNH) was calculated from the adjusted risk difference between exposed and never exposed infants. For this analysis, the adjusted frequencies of NICU admission, PPHN and neurological disorders amongst the exposed infants were calculated from the results of the multivariate risk analyses for these outcomes, to adjust for the risk of the underlying maternal condition. The statistical analyses were conducted by using SPSS version 27 (IBM SPSS Statistics, IBM Corporation, Armonk, NY).

Results

Among the included infants, 2677 (0.2%) were exposed to antipsychotics during pregnancy, and 34 492 (2.6%) had mothers treated with antipsychotics before or after but not during the

For peer review only - http://bmjopen.bmj.com/site/about/guidelines.xhtml

pregnancy. The study outline is presented as a flowchart in Figure 1. The most prescribed antipsychotics in the pregnant population during the study period were quetiapine (1021 exposures), olanzapine (771 exposures) and aripiprazole (334 exposures), all second-generation antipsychotics. The most prescribed first-generation antipsychotic was levomepromazine with 251 exposures. (Supplemental Table 1)

Table 1 summarizes the background characteristics of the included infants and their mothers. The infants exposed to antipsychotics were more likely to be born with a caesarean section, to be small for gestational age, and to be born preterm, and their mothers were more likely to smoke, be overweight or obese, not be living with the father of the child and to be using concomitant neurotropic medications, compared to both unexposed infants and infants to mothers treated with antipsychotics before and/or after the pregnancy. In the exposed group, 40.7% of the mothers used any other neurotropic drug listed in table 1 during pregnancy, compared to 2.7% in the unexposed group. No statistically significant difference between the groups was found in the incidence of preeclampsia, the infant being large for gestational age, perinatal death, or infant sex.

The risk of being admitted to neonatal care was increased for infants exposed to antipsychotics in early pregnancy only compared to non-exposed peers, adjusted RR [aRR] 1.5, 95% confidence interval [CI] 1.3-1.7. Exposure in late pregnancy increased the risk further, with aRR 1.8, 95% CI 1.6-2.0, adjusted for concomitant use of other psychotropic drugs. In sensitivity analyses, the relative risk for neonatal care admission for infants exposed to antipsychotics during late pregnancy vs non-exposed was instead stratified by use of other psychotropic drugs and were found to be similar to the previously obtained aRRs. The aRRs were 1.7, 95% CI 1.4-2.0 and 2.0, 95% CI 1.8-2.3 for children exposed, or not exposed to other psychotropic drugs, respectively. The risk of being admitted to NICU was similar for infants exposed to first- and second-generation antipsychotics (Table 2). When compared to

For peer review only - http://bmjopen.bmj.com/site/about/guidelines.xhtml

infants born to women treated with antipsychotics before or after but not during pregnancy, the risk increase for admission to NICU was lower but still statistically significant. Additional adjustment for gestational age and Z-score for infant weight did not markedly reduce the risk for being admitted to NICU for any group of infants. (Supplemental Table 2). The NNH for NICU admission compared to the unexposed infants adjusted for maternal factors was 18.

The neonatal disorders with increased risks associated with antipsychotics exposure were withdrawal symptoms from therapeutic drugs (aRR 17.7, 95% CI 9.6-32.6), neurological disorders (aRR 3.4, 95% CI 2.5-4.7) and PPHN (aRR 2.1, 95% CI 1.4-3.1) when compared to the unexposed infants. However, the absolute frequencies of these diagnoses were still less than two percent amongst the exposed infants (Table 3). A sensitivity analysis on PPHN only including term infants born at gestational week 37 or later did not change the risk estimates. Adjusted NNH for neurological disorders was 139 and for PPHN 227, when compared to the unexposed infants.

The most frequent outcomes affecting 5-7% of the exposed infants were respiratory disorders, hyperbilirubinemia, and hypoglycaemia. The adjusted risks for these conditions were moderately increased for exposed infants, slightly lower when compared to the control group exposed before/after pregnancy than in comparison to the unexposed infants. (Table 3) Additional adjustment for fetal factors including prematurity did not markedly change these risks. (Supplemental table 3) No statistically significant differences between the groups were found for respiratory distress syndrome, ventilator treatment, any malformations or heart malformations.

To grade the severity of the neonatal morbidity after exposure to antipsychotics, length of stay was calculated for the full-term infants admitted to NICU. (Table 4) Median length of stay in infants exposed to antipsychotics during pregnancy was 5 days, similar to the infants not exposed. However, when corrected for maternal factors, the exposed infants had slightly but

For peer review only - http://bmjopen.bmj.com/site/about/guidelines.xhtml

statistically significantly longer length of stay at NICU when compared to infants to mothers never exposed to antipsychotics. For full-term infants with PPHN and/or neurological disorders, the length of stay at NICU was slightly shorter than for the unexposed infants.

Discussion

Neonatal illness and the risk for admission to NICU were found to be increased amongst the exposed infants, at similar levels for both first- and second-generation antipsychotics. The risk of needing neonatal care was further increased after exposure in late pregnancy. The equal or slightly longer length of stay at NICU in exposed infants compared to never exposed infants indicate that these admissions reflect real neonatal illness and are not only a cautionary measure due to the exposure. The risk for PPHN was also clearly increased for the exposed infants, which has to our knowledge not been previously described related to antipsychotics.

Our results provide a detailed description of the neonatal morbidity that has not been published before in a study of this size. It is likely that the larger cohort size in this study enables studying the rarer neonatal outcomes including the two overlapping diagnoses of neurological outcomes and drug withdrawal symptoms. These outcomes have not previously been shown to be increased amongst exposed infants, but in this study, they are clearly increased even after adjustments for maternal factors. [22 23] An increase in neonatal morbidity after use of antipsychotics in late pregnancy that we found has also been observed for selective serotonin reuptake inhibitors (SSRIs).[36] Considering the mechanisms of these drugs in the serotonergic and dopaminergic pathways of the central nervous system there is likely to be a biological, drug-related explanation behind this.[37] However, the causality of this connection remains to be clarified.

The Swedish nationwide registers have some limitations regarding data on the drug exposure, that are based on filled prescriptions (PDR) and patient interviews (MBR) and not containing

For peer review only - http://bmjopen.bmj.com/site/about/guidelines.xhtml

information on to what extent the women took the drugs. It is likely that some degree of exposure misclassification exists. Since information on drug intake was collected before the outcomes were known, it is unlikely the exposure misclassification has seriously biased the results. Even with these draw-backs, we consider the results from this study to be well generalizable to other cohorts, due to the nationwide heterogeneous cohort.

The risk estimates for NICU admission and the studied neonatal disorders decreased but remained significant when exposed infants were compared to infants to mothers treated with antipsychotics before or after pregnancy. This indicates that a part of, but not all, the risk-increase for neonatal morbidity seen in the exposed infants is likely to be attributable to underlying factors rather than the drug treatment. However, as the results remained significant after adjustments, it is likely, that there is also a true risk for neonatal morbidity connected to the antipsychotic drug treatment, especially after exposure in late pregnancy. Interestingly, the risk for being admitted to NICU was similar for infants to mothers with antipsychotic treatment in monotherapy and infants whose mothers had used other neurotropic drugs together with antipsychotics during pregnancy. This indicates, that the connection between antipsychotic exposure during late pregnancy and admission to NICU cannot be explained by the high percentage of mothers with concomitant medications in the exposed group. For policymakers it is important to consider the needs for a well-developed and structured healthcare for women with psychiatric disorders and their infants. For the clinicians, it is also important to acknowledge the crude risk estimates of this study, as they reflect the true risks for this group of patients, burdened with comorbidities. Whether there is a dose-response relationship to these effects remains to be studied.

The risk-increase for PPHN that was found could be explained by poor adaptation in these infants due to the neurological effects of the drugs, leading to both respiratory disorders and down the line even persistent pulmonary hypertension. Over half of the exposures in our data

For peer review only - http://bmjopen.bmj.com/site/about/guidelines.xhtml

set consist of olanzapine and quetiapine, two substances affecting serotonergic as well as dopaminergic receptors.[38] A similar, but milder, correlation to PPHN has also been shown for prenatal exposure for SSRIs.[36 39] Therefore we speculate that an effect on the serotonergic system might partly explain the increased risk of PPHN. The length of stay at NICU was shorter in exposed full-term infants with PPHN than in their unexposed peers, indicating that PPHN connected to fetal exposure to antipsychotics could be less severe than PPHN secondary to other causes. Some of the risk-increase could also be due to over-diagnostics of PPHN amongst exposed infants with respiratory disorders. The risk for neurological outcomes seems increased on group level, but whether it is a causal effect of the antipsychotic drugs and what neurological outcomes these infants have increased risks for is yet to be determined. The length of stay at NICU was slightly shorter in infants exposed to antipsychotics with neurological disorders than unexposed infants, but still rather long, nine days in median, indicating that even though some over diagnostics of the exposed infants might be present, the exposed infants did require more than just mere observation. Further studies with larger cohorts are needed to separate the risks for the different antipsychotic drugs and the different neurological outcomes from each other.

Conclusion

Fetal exposure to antipsychotics is associated with an increased risk for neonatal morbidity. The risk seems similar for infants exposed to first- and second- generation antipsychotics, and more pronounced after exposure in late pregnancy, and the increased risks for PPHN and neurological outcomes require further studies. In general, the neonatal disorders seen in exposed infants seem transient and predominantly mild, and these findings do not warrant discontinuation of a necessary treatment but rather increased monitoring of these infants postpartum.

For peer review only - http://bmjopen.bmj.com/site/about/guidelines.xhtml

Author contributions

All authors planned and designed the study together and approved the final manuscript as submitted and agree to be accountable for all aspects of the work.

Prof Källén was responsible for the data collection. Dr Heinonen drafted the initial manuscript. Prof. Källén and drs Forsberg, Nörby and Wide reviewed and revised the manuscript.

Competing interests

The authors have no conflicts of interest relevant to this article to disclose.

Funding

This work was supported by the Swedish Research Council, grant number 521-2012-3466, (EH, KW, LF), Stockholm County Council (ALF) with grant number 573301 (EH, KW, LF). The funders did not participate in the work.

Availability of data and material

Individual data are protected by the General Data Protection Regulation in EU (GDPR). All data is stored and accessible with permission only in a data repository at The Swedish National Board of Health and Welfare.

Acknowledgements

None

References

1. Alexander GC, Gallagher SA, Mascola A, et al. Increasing off-label use of antipsychotic medications in the United States, 1995-2008. *Pharmacoepidemiology and Drug Safety* 2011;20(2):177-84. doi: 10.1002/pds.2082
2. Stephenson CP, Karanges E, McGregor IS. Trends in the utilisation of psychotropic medications in Australia from 2000 to 2011. *Australian & New Zealand Journal of Psychiatry* 2013;47(1):74-87. doi: 10.1177/0004867412466595
3. Galbally M, Snellen M, Power J. Antipsychotic drugs in pregnancy: a review of their maternal and fetal effects. *Therapeutic Advances in Drug Safety* 2014;5(2):100-09. doi: 10.1177/2042098614522682

For peer review only - http://bmjopen.bmj.com/site/about/guidelines.xhtml

4. Hatters Friedman S, Moller-Olsen C, Prakash C, et al. Atypical antipsychotic use and outcomes in an urban maternal mental health service. *The International Journal of Psychiatry in Medicine* 2016;51(6):521-33. doi: 10.1177/0091217417696739
5. Ellfolk M, Leinonen MK, Gissler M, et al. Second-generation antipsychotics and pregnancy complications. *European Journal of Clinical Pharmacology* 2020;76(1):107-15. doi: 10.1007/s00228-019-02769-z
6. Toh S, Li Q, Cheetham TC, et al. Prevalence and trends in the use of antipsychotic medications during pregnancy in the U.S., 2001–2007: a population-based study of 585,615 deliveries. *Archives of Women's Mental Health* 2013;16(2):149-57. doi: 10.1007/s00737-013-0330-6
7. Huybrechts KF, Hernandez-Diaz S, Patorno E, et al. Antipsychotic Use in Pregnancy and the Risk for Congenital Malformations. *JAMA psychiatry* 2016;73(9):938-46. doi: 10.1001/jamapsychiatry.2016.1520
8. Kulkarni J, Storch A, Baraniuk A, et al. Antipsychotic use in pregnancy. *Expert Opinion on Pharmacotherapy* 2015;16(9):1335-45. doi: 10.1517/14656566.2015.1041501
9. Habermann F, Fritzsche J, Fuhlbruck F, et al. Atypical antipsychotic drugs and pregnancy outcome: a prospective, cohort study. *J Clin Psychopharmacol* 2013;33(4):453-62. doi: 10.1097/JCP.0b013e318295fe12 [published Online First: 2013/06/15]
10. McKenna K, Koren G, Tetelbaum M, et al. Pregnancy outcome of women using atypical antipsychotic drugs: a prospective comparative study. *J Clin Psychiatry* 2005;66(4):444-9; quiz 546.
11. Reis M, Kallen B. Maternal use of antipsychotics in early pregnancy and delivery outcome. *J Clin Psychopharmacol* 2008;28(3):279-88. doi: 10.1097/JCP.0b013e318172b8d5 [published Online First: 2008/05/16]
12. Kulkarni J, Worsley R, Gilbert H, et al. A prospective cohort study of antipsychotic medications in pregnancy: the first 147 pregnancies and 100 one year old babies. *PLoS One* 2014;9(5):e94788. doi: 10.1371/journal.pone.0094788
13. Einarson A, Boskovic R. Use and Safety of Antipsychotic Drugs During Pregnancy. *Journal of Psychiatric Practice* 2009;15(3):183-92. doi: 10.1097/01.pra.0000351878.45260.94
14. Gentile S, Fusco ML. Schizophrenia and motherhood. *Psychiatry and Clinical Neurosciences* 2019;73(7):376-85. doi: 10.1111/pcn.12856
15. Gentile S. Antipsychotic therapy during early and late pregnancy. A systematic review. *Schizophr Bull* 2010;36(3):518-44. doi: 10.1093/schbul/sbn107
16. Kallen B, Borg N, Reis M. The use of central nervous system active drugs during pregnancy. *Pharmaceuticals* 2013;6(10):1221-86. doi: 10.3390/ph6101221
17. Ozdemir AK, Pak SC, Canan F, et al. Paliperidone palmitate use in pregnancy in a woman with schizophrenia. *Arch Womens Ment Health* 2015;18(5):739-40. doi: 10.1007/s00737-014-0496-6
18. Viguera AC, Whitfield T, Baldessarini RJ, et al. Risk of Recurrence in Women With Bipolar Disorder During Pregnancy: Prospective Study of Mood Stabilizer Discontinuation. *American Journal of Psychiatry* 2007;164(12):1817-24. doi: 10.1176/appi.ajp.2007.06101639
19. Newport DJ, Calamaras MR, DeVane CL, et al. Atypical antipsychotic administration during late pregnancy: placental passage and obstetrical outcomes. *Am J Psychiatry* 2007;164(8):1214-20. doi: 10.1176/appi.ajp.2007.06111886
20. Sadowski A, Todorow M, Yazdani Brojeni P, et al. Pregnancy outcomes following maternal exposure to second-generation antipsychotics given with other psychotropic drugs: a cohort study. *BMJ Open* 2013;3(7) doi: 10.1136/bmjopen-2013-003062
21. Terrana N, Koren G, Pivovarov J, et al. Pregnancy Outcomes Following In Utero Exposure to Second-Generation Antipsychotics: A Systematic Review and Meta-Analysis. *J Clin Psychopharmacol* 2015;35(5):559-65. doi: 10.1097/JCP.0000000000000391 [published Online First: 2015/08/15]

For peer review only - http://bmjopen.bmj.com/site/about/guidelines.xhtml

22. Vigod SN, Gomes T, Wilton AS, et al. Antipsychotic drug use in pregnancy: high dimensional, propensity matched, population based cohort study. *BMJ* 2015;350:h2298. doi: 10.1136/bmj.h2298
23. Petersen I, McCrea RL, Sammon CJ, et al. Risks and benefits of psychotropic medication in pregnancy: cohort studies based on UK electronic primary care health records. *Health Technol Assess* 2016;20(23):1-176. doi: 10.3310/hta20230 [published Online First: 2016/04/01]
24. Frayne J, Nguyen T, Bennett K, et al. The effects of gestational use of antidepressants and antipsychotics on neonatal outcomes for women with severe mental illness. *Australian and New Zealand Journal of Obstetrics and Gynaecology* 2017;57(5):526-32. doi: 10.1111/ajo.12621
25. Jablensky AV, Morgan V, Zubrick SR, et al. Pregnancy, delivery, and neonatal complications in a population cohort of women with schizophrenia and major affective disorders. *Am J Psychiatry* 2005;162(1):79-91. doi: 10.1176/appi.ajp.162.1.79 [published Online First: 2004/12/31]
26. Boden R, Lundgren M, Brandt L, et al. Risks of adverse pregnancy and birth outcomes in women treated or not treated with mood stabilisers for bipolar disorder: population based cohort study. *BMJ* 2012;345:e7085. doi: 10.1136/bmj.e7085 [published Online First: 2012/11/10]
27. Abel K. HL. Schizophrenia, psychopharamacology and pregnancy. . Galbally M, Snellen M, Lewis A (eds), Psychopharmacology and Pregnancy – Treatment Efficacy, Risks, and Guidelines. Heidelberg: Springer 2014.
28. Epidemiology. Cf. The Swedish Medical Birth Register: a summary of content and quality. [Available from: https://www.socialstyrelsen.se/globalassets/sharepoint-dokument/artikelkatalog/ovrigt/2003-112-3_20031123.pdf accessed November 9 2020.
29. Wettermark B, Hammar N, Michaelfored C, et al. The new Swedish Prescribed Drug Register—Opportunities for pharmacoepidemiological research and experience from the first six months. *Pharmacoepidemiology and Drug Safety* 2007;16(7):726-35. doi: 10.1002/pds.1294
30. Register. SNQ. [accessed November 9 2020.
31. Molin J. A regional perinatal database in southern Sweden--a basis for quality assurance in obstetrics and neonatology. *Acta Obstet Gynecol Scand Suppl* 1997;164:37-39.
32. Ornoy A. Valproic acid in pregnancy: How much are we endangering the embryo and fetus? *Reproductive Toxicology* 2009;28(1):1-10. doi: 10.1016/j.reprotox.2009.02.014
33. Levels of Neonatal Care. *PEDIATRICS* 2012;130(3):587-97. doi: 10.1542/peds.2012-1999
34. Maršál K, Persson PH, Larsen T, et al. Intrauterine growth curves based on ultrasonically estimated foetal weights. *Acta Paediatrica* 1996;85(7):843-48. doi: 10.1111/j.1651-2227.1996.tb14164.x
35. von Elm E, Altman DG, Egger M, et al. The Strengthening the Reporting of Observational Studies in Epidemiology (STROBE) statement: guidelines for reporting observational studies. *J Clin Epidemiol* 2008;61(4):344-9. doi: 10.1016/j.jclinepi.2007.11.008 [published Online First: 2008/03/04]
36. Norby U, Forsberg L, Wide K, et al. Neonatal Morbidity After Maternal Use of Antidepressant Drugs During Pregnancy. *Pediatrics* 2016;138(5) doi: 10.1542/peds.2016-0181
37. Ritter J. Rang and Dale's pharmacology (Ninth edition.). Edinburgh: Elsevier 2020:592-97.
38. Grinchii D, Dremencov E. Mechanism of Action of Atypical Antipsychotic Drugs in Mood Disorders. *International Journal of Molecular Sciences* 2020;21(24):9532. doi: 10.3390/ijms21249532
39. Kieler H, Artama M, Engeland A, et al. Selective serotonin reuptake inhibitors during pregnancy and risk of persistent pulmonary hypertension in the newborn: population based cohort study from the five Nordic countries. *BMJ* 2012;344:d8012. doi: 10.1136/bmj.d8012 [published Online First: 2012/01/14]

For peer review only - http://bmjopen.bmj.com/site/about/guidelines.xhtml

	Anti-psychotics during pregnancy n=2677 n (%)	Anti-psychotics before or after but not during pregnancy n=34 492 n (%)	No anti-psychotic use[1] n=1 262 047 n (%)	Anti-psychotics during pregnancy vs anti-psychotics before/after pregnancy p	Anti-psychotics during pregnancy vs no anti-psychotic use p	Anti-psychotics before/after pregnancy vs no anti-psychotic use p
**Year of Child Birth**				<0.001	<0.001	<0.001
2006-2010	1013 (37.8)	19 279 (55.9)	611 663 (48.3)			
2011-2017	1664 (62.2)	15 213 (44.1)	650 384 (51.5)			
**Maternal Age, y**				<0.001	0.020	<0.001
<20	70 (2.6)	1059 (3.1)	25 554 (2.0)			
20-35	1883 (70.3)	26 638 (77.2)	964 380 (76.4)			
35+	724 (27)	6795 (19.7)	272 113 (21.6)			
**Parity**				<0.001	<0.001	<0.001
Primipara	1891 (70.6)	20 727 (60.1)	797 603 (63.2)			
Multipara	786 (29.4)	13 764 (39.9)	456 556 (36.2)			
**BMI in Early Pregnancy**				<0.001	<0.001	<0.001
<24.9	1066 (39.8)	17 284 (50.1)	717 953 (56.8)			
25-29.9	772 (28.8)	8516 (24.7)	298162 (23.6)			
>=30	618 (23.1)	5723 (16.6)	147 627 (11.7)			
BMI Unknown	221 (8.3)	2969 (8.6)	98 302 (7.8)			
**Smoking in Early Pregnancy**				<0.001	<0.001	<0.001
No	1818 (67.9)	26 774 (77.6)	1 128 043 (89.4)			
Yes	733 (27.4)	5939 (17.2)	67 348 (5.3)			
Missing information	126 (4.7)	1779 (5.2)	66 656 (5.3)			
**Maternal Country of Birth**				<0.001	0.209	<0.001
Sweden	2018 (75.4)	27 655 (80.2)	928 818 (73.6)			
Other nordic	123 (4.6)	946 (2.7)	51 223 (4.1)			
Non-nordic	525 (19.6)	5844 (16.9)	257 259 (20.4)			
**Maternal Cohabitation**				<0.001	<0.001	<0.001
Not living with father of child	657 (24.5)	4824 (14.0)	75 451 (6.0)			
**Concomitant Neurotropic Drugs**						
Lithium (N05A N01)	124 (4.6)	292 (0.8)	2 (0.0)	<0.001	<0.001	<0.001
Opioids (N02A)	44 (1.6)	604 (1.8)	4748 (0.4)	0.680	<0.001	<0.001
Antiepileptics (N03A)	215 (8.0)	725 (2.1)	2989 (0.2)	<0.001	<0.001	<0.001
Antidepressants (N06A)	755 (28.2)	4409 (12.8)	23 926 (1.9)	<0.001	<0.001	<0.001
Psychostimulants (N06B)	88 (3.3)	328 (1.0)	790 (0.1)	<0.001	<0.001	<0.001
Anxiolytics & sedatives (N05B, C)	436 (16.3)	1429 (4.1)	4620 (0.4)	<0.001	<0.001	<0.001
Milder anxiolytics & antiemetics[2]	366 (13.7)	2516 (7.3)	24 422 (1.0)	<0.001	<0.001	<0.001
**Pregnancy Complications**						
Gestational Diabetes	76 (2.8)	493 (1.4)	14 979 (1.2)	<0.001	<0.001	<0.001
Preeclampsia	25 (0.9)	346 (1.0)	10 345 (0.8)	0.729	0.512	<0.001
Cesarean Section	687 (25.7)	7178 (20.8)	206 586 (16.4)	<0.001	<0.001	<0.001
Preterm birth	259 (9.7)	2471 (7.2)	67 637 (5.3)	<0.001	<0.001	<0.001
Small for Gestational Age	104 (3.9)	929 (2.7)	29 182 (2.3)	<0.001	<0.001	<0.001
Large for Gestational Age	126 (4.7)	1410 (4.1)	50 421 (4.0)	0.121	0.060	0.386
APGAR 5min <7	90 (3.4)	737 (2.1)	17 244 (1.4)	<0.001	<0.001	<0.001
Perinatal Death	16 (0.6)	162 (0.5)	5057 (0.4)	0.357	0.108	0.046
**Infant Sex**				0.777	0.610	0.454
Male	1365 (51.0)	17 660 (51.2)	644 906 (51.1)			
Female	1312 (49.0)	16 832 (48.8)	617 141 (48.9)			

Table 1. Background Characteristics of the Study Population. [1] No use during the entire study period. [2] Including Promethazine (R06AD02, R06AD52), Alimemazine (R06AD01), Dixyrazine (N05A B01), Prochlorperazine (N05A B04) and Melperone (N05A D03)

For peer review only

For peer review only - http://bmjopen.bmj.com/site/about/guidelines.xhtml

	NICU Admissions n (%)	Exposed vs Never exposed				Exposed Infants vs Infants to Mothers Treated with Antipsychotics Before or After the Pregnancy			
		Crude RR	95% CI	Adj. RR	95% CI	Crude RR	95% CI	Adj. RR	95% CI
**Exposed early pregnancy only**									
Any Antipsychotics, n=1454	251 (17.3)	2.2	2.0-2.5	1.5	1.3-1.7	1.5	1.3-1.7	1.3	1.1-1.4
First-generation, n=431	76 (17.6)	2.2	1.7-2.7	1.5	1.2-1.9	1.5	1.2-1.8	1.3	1.0-1.6
Second-generation, n=1166	212 (18.2)	2.2	2.0-2.6	1.5	1.3-1.7	1.5	1.3-1.7	1.3	1.1-1.4
**Exposed in late pregnancy**									
Any Antipsychotics, n=1223	265 (21.7)	2.8	2.5-3.1	1.8	1.6-2.0	1.9	1.7-2.1	1.5	1.4-1.7
First-generation, n=297	73 (24.6)	3.1	2.6-3.8	2.1	1.7-2.5	2.1	1.7-2.6	1.7	1.4-2.1
Second-generation, n=972	208(21.4)	2.7	2.4-3.1	1.8	1.6-2.0	1.9	1.6-2.1	1.5	1.3-1.7
**Never exposed, n=1262 047**	98976 (7.8)	REFERENCE GROUP 1							
**Exposed before or after but not during pregnancy, n=34 492**	4010 (11.6)	REFERENCE GROUP 2							

Table 2. Risk ratio (RR) for admission to neonatal intensive care unit (NICU) compared with non-exposed infants and with infants to mothers using antipsychotics before and/or after but not during the current pregnancy. Adjusted for: Primipara, Age, Body Mass Index (BMI), Smoking, Cesarean section, Concurrent neurotropic drugs.

For peer review only

Infant outcomes	Exposed during pregnancy N=2677	Exposed before/after but not during pregnancy N=34 492	Never exposed N=1 262 047	Exposed vs Never exposed				Exposed Infants vs Infants to Mothers Treated with Antipsychotics Before or After the Pregnancy			
	n (%)	n (%)	n (%)	Crude RR	95% CI	Adj. RR	95% CI	Crude RR	95% CI	Adj. RR	95% CI
**Any birth defect** [a]	64/2378 (2.7)	27122 (2.1)	822 (2.4)	1.3	1.0-1.6	1.1	0.8-1.4	1.1	0.9-1.5	1.1	0.8-1.4
Any heart defect [a]	23/2378 (1.0)	9644 (0.8)	304 (0.9)	1.3	0.8-1.9	1.1	0.7-1.6	1.1	0.7-1.7	1.0	0.7-1.6
**Admission to NICU**	516 (19.3)	4010 (11.6)	98976 (7.8)	2.5	2.3-2.7	1.7	1.6-1.8	1.7	1.5-1.8	1.4	1.3-1.5
**Respiratory symptoms**											
Transient tachypnea of the newborn	185 (6.9)	1406 (4.1)	34204 (2.7)	2.5	2.2-2.9	1.6	1.4-1.9	1.7	1.5-2.0	1.4	1.2-1.7
Persistent pulmonary hypertension	27 (1.0)	209 (0.6)	4581 (0.4)	2.8	1.9-4.1	2.1	1.4-3.1	1.7	1.1-2.5	1.5	1.0-2.2
Respiratory distress syndrome	21 (0.8)	296 (0.9)	6671 (0.5)	1.5	1.0-2.3	1.0	0.7-1.6	0.9	0.6-1.4	0.9	0.6-1.4
**Respiratory treatment**											
CPAP	168 (6.3)	1276 (3.7)	29977 (2.4)	2.7	2.3-3.1	1.7	1.4-2.0	1.7	1.5-2.0	1.5	1.2-1.7
Ventilator treatment	22 (0.8)	299 (0.9)	6811 (0.5)	1.5	1.0-2.3	1.1	0.7-1.6	1.0	0.6-1.5	0.9	0.6-1.4
**Hyperbilirubinemia**	169 (6.3)	1819 (5.3)	54563 (4.3)	1.5	1.3-1.7	1.3	1.1-1.5	1.2	1.0-1.4[b]	1.1	1.0-1.3
**Hypoglycemia**	133 (5.0)	1090 (3.2)	28117 (2.2)	2.2	1.9-2.6	1.4	1.2-1.7	1.6	1.3-1.9	1.3	1.1-1.5
**Feeding difficulties**	75 (2.8)	558 (1.6)	14147 (1.1)	2.5	2.0-3.1	1.8	1.4-2.3	1.7	1.4-2.2	1.6	1.2-2.0
**Neurological disorders**	47 (1.8)	186 (0.5)	3821 (0.3)	5.8	4.4-7.7	3.4	2.5-4.7	3.3	2.4-4.5	2.3	1.6-3.3
**Withdrawal symptoms from therapeutic drugs**	34 (1.3)	82 (0.2)	153 (0.0)	105	72.4-152	17.7	9.6-32.6	5.3	3.6-8.0	3.3	2.1-5.3

For peer review only - http://bmjopen.bmj.com/site/about/guidelines.xhtml

Table 3. Risk ratio (RR) for birth defects and neonatal morbidity after exposure to antipsychotics. NICU = Neonatal intensive Care Unit. CPAP = Continuous positive airway pressure. Adjusted for: Primipara, Age, Body Mass Index (BMI), Smoking, Cesarean section, Concurrent neurotropic drugs. [a] Exposures are only counted if present in early pregnancy (n=2378). [b] $p < 0.05$

Length of stay amongst term infants admitted to NICU (days)	All term infants		Infants with diagnosis of PPHN		Infants with neurological disorders	
	n	Median (IQ-range)	n	Median (IQ-range)	n	Median (IQ-range)
Exposed	336	5 (3-9)	12	8 (3-9)	34	9 (6-11)
Exposed before/after pregnancy	2380	5 (2-8)	80	9 (6-15)	129	11 (7-18)
Not exposed	59 439	5 (2-8)	1894	9 (5-17)	2698	11 (7-18)

Table 4. Length of Stay at Neonatal Intensive Care Unit (NICU) amongst term infants presented in median days and interquartile (IQ)-ranges. Presented for all term infants, infants with persistent pulmonary hypertension (PPHN) and infants with neurological disorder.

Figure 1. Flowchart over the study design. 1) Women with pre-pregnancy Diabetes mellitus. 2) Use of Valproate during pregnancy. NICU = Neonatal Intensive Care Unit.

For peer review only

For peer review only - http://bmjopen.bmj.com/site/about/guidelines.xhtml

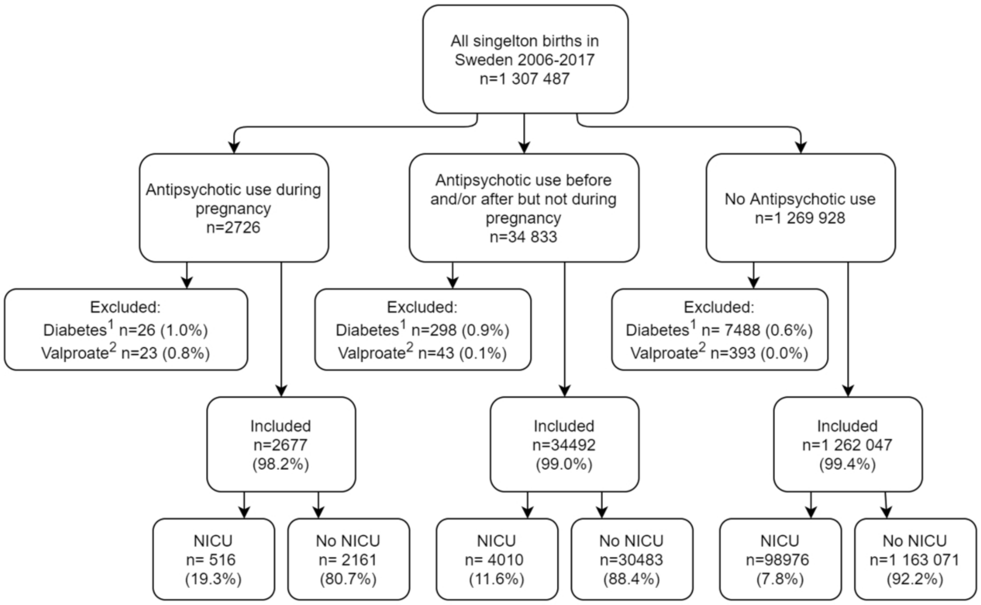

Figure 1. Flowchart over the study design. 1) Women with pre-pregnancy Diabetes mellitus. 2) Use of Valproate during pregnancy. NICU = Neonatal Intensive Care Unit.

522x325mm (300 x 300 DPI)

Supplemental material for: Heinonen et al. Neonatal morbidity after fetal exposure to antipsychotics - a national register-based study

EXPOSURE	n
**First-generation antipsychotics**	
Levomepromazine	251
Flupentixol	165
Haloperidol	162
Perphenazine	93
Zuclopenthixol	75
Chlorprothixene	45
Chlorpromazine	4
Pimozide	4
Fluphenazine	3
Thioridazine	1
**Second-generation antipsychotics**	
Quetiapine	1026
Olanzapine	771
Aripiprazole	334
Risperidone	191
Ziprasidone	34
Clozapine	29
Paliperidone	9
Sertrindole	2
**TOTAL**	3199

Supplemental Table 1. Prescriptions of antipsychotics during pregnancy 2006-2017.

For peer review only

NICU-admissions	Exposed vs Never exposed						Exposed Infants vs Infants to Mothers Treated with Antipsychotics Before or After the Pregnancy					
	Crude		Adjusted for Maternal Factors Only		Adjusted for Maternal and Fetal Factors		Crude		Adjusted for Maternal Factors Only		Adjusted for Maternal and Fetal Factors	
	RR	95% CI	RR	95% CI	RR	95% CI	RR	95% CI	RR	95% CI	RR	95% CI
**Exposed early pregnancy only**												
Any Antipsychotics, n=1454	2.2	2.0-2.5	1.5	1.3-1.7	1.3	1.1-1.6	1.5	1.3-1.7	1.3	1.1-1.4	1.2	1.0-1.4
F-GA, n=431	2.2	1.7-2.7	1.5	1.2-1.9	1.5	1.2-1.8	1.5	1.2-1.8	1.3	1.0-1.6	1.3	1.0-1.6*
S-GA, n=1166	2.3	2.0-2.6	1.5	1.3-1.7	1.3	1.0-1.7*	1.5	1.3-1.7	1.3	1.1-1.4	1.2	0.9-1.4
**Exposed in late pregnancy**												
Any Antipsychotics, n=1223	2.8	2.5-3.1	1.8	1.6-2.0	1.8	1.6-2.0	1.9	1.7-2.1	1.5	1.4-1.7	1.5	1.4-1.7
F-GA, n=297	3.1	2.6-3.8	2.1	1.7-2.5	2.1	1.7-2.5	2.1	1.7-2.6	1.7	1.4-2.1	1.7	1.4-2.0
S-GA, n=972	2.7	2.4-3.1	1.8	1.6-2.0	1.8	1.6-2.0	1.9	1.6-2.1	1.5	1.3-1.7	1.5	1.3-1.7
**Never exposed, n=1262 047**	REFERENCE GROUP 1											
**Exposed before or after but not during pregnancy, n=34 492**	REFERENCE GROUP 2											

Supplemental table 2. Risk (RR) for admission to neonatal intensive care unit (NICU) compared with non-exposed infants and with infants to mothers using antipsychotics before and/or after but not during the current pregnancy. F-GA = First-generation Antipsychotics, S-GA = Second-generation Antipsychotics. Maternal factors: Primipara, Age, Body Mass Index (BMI), Smoking, Cesarean section, Concurrent neurotropic drugs. Fetal factors: Gestational Age, Z-score. * p <0.05

For peer review only - http://bmjopen.bmj.com/site/about/guidelines.xhtml

Neonatal outcomes	Exposed vs Never exposed						Exposed Infants vs Infants to Mothers Treated with Antipsychotics Before or After the Pregnancy					
	Crude		Adjusted for Maternal Factors Only		Adjusted for Maternal and Fetal Factors		Crude		Adjusted for Maternal Factors Only		Adjusted for Maternal and Fetal Factors	
	RR	95% CI	RR	95% CI	RR	95% CI	RR	95% CI	RR	95% CI	RR	95% CI
**Admission to NICU**	2.5	2.3-2.7	1.7	1.6-1.8	1.6	1.4-1.6	1.7	1.5-1.8	1.4	1.3-1.5	1.4	1.2-1.5
**Respiratory symptoms**												
Transient tachypnea of the newborn	2.5	2.2-2.9	1.6	1.4-1.9	1.5	1.3-1.8	1.7	1.5-2.0	1.4	1.2-1.7	1.4	1.2-1.6
Persistent pulmonary hypertension	2.8	1.9-4.1	2.1	1.4-3.1	1.5	0.9-2.6	1.7	1.1-2.5	1.5	1.0-2.2	1.2	0.7-2.2
Respiratory distress syndrome	1.5	1.0-2.3	1.0	0.7-1.6	0.7	0.3-1.5	0.9	0.6-1.4	0.9	0.6-1.4	0.6	0.3-1.3
**Respiratory treatment**												
CPAP	2.7	2.3-3.1	1.7	1.4-2.0	1.5	1.1-2.0	1.7	1.5-2.0	1.5	1.2-1.7	1.3	1.0-1.7
Ventilator treatment	1.5	1.0-2.3	1.1	0.7-1.6	0.8	0.4-1.5	1.0	0.6-1.5	0.9	0.6-1.4	0.7	0.3-1.4
**Hyperbilirubinemia**	1.5	1.3-1.7	1.3	1.1-1.5	1.2	0.9-1.4	1.2	1.0-1.4*	1.1	1.0-1.3	1.1	0.9-1.3
**Hypoglycemia**	2.2	1.9-2.6	1.4	1.2-1.7	1.3	1.1-1.6	1.6	1.3-1.9	1.3	1.1-1.5	1.2	1.0-1.5*
**Feeding difficulties**	2.5	2.0-3.1	1.8	1.4-2.3	1.7	1.3-2.2	1.7	1.4-2.2	1.6	1.2-2.0	1.5	1.1-1.9
**Neurological disorders**	5.8	4.4-7.7	3.4	2.5-4.7	3.3	2.4-4.6	3.3	2.4-4.5	2.3	1.6-3.3	2.3	1.6-3.3
**Withdrawal symptoms from therapeutic drugs**	105	72.4-152	17.7	9.6-32.6	17.3	9.4-31.7	5.3	3.6-8.0	3.3	2.1-5.3	3.4	2.1-5.3

Supplemental table 3. Risk (RR) for neonatal morbidity after exposure to antipsychotics. NICU = Neonatal intensive Care Unit. CPAP = Continuous positive airway pressure. Maternal factors: Primipara, Age, Body Mass Index (BMI), Smoking, Cesarean section, Concurrent neurotropic drugs. Fetal factors: Gestational Age, Z-score. * $p < 0.05$

For peer review only

Reporting checklist for cohort study.

Based on the STROBE cohort guidelines.

Instructions to authors

Complete this checklist by entering the page numbers from your manuscript where readers will find each of the items listed below.

Your article may not currently address all the items on the checklist. Please modify your text to include the missing information. If you are certain that an item does not apply, please write "n/a" and provide a short explanation.

Upload your completed checklist as an extra file when you submit to a journal.

In your methods section, say that you used the STROBE cohortreporting guidelines, and cite them as:

von Elm E, Altman DG, Egger M, Pocock SJ, Gotzsche PC, Vandenbroucke JP. The Strengthening the Reporting of Observational Studies in Epidemiology (STROBE) Statement: guidelines for reporting observational studies.

For peer review only

		Reporting Item	Page Number
**Title and abstract**			
Title	#1a	Indicate the study’s design with a commonly used term in the title or the abstract	1
Abstract	#1b	Provide in the abstract an informative and balanced	2

		summary of what was done and what was found	
**Introduction**			
Background / rationale	#2	Explain the scientific background and rationale for the investigation being reported	3
Objectives	#3	State specific objectives, including any prespecified hypotheses	3
**Methods**			
Study design	#4	Present key elements of study design early in the paper	4
Setting	#5	Describe the setting, locations, and relevant dates, including periods of recruitment, exposure, follow-up, and data collection	4
Eligibility criteria	#6a	Give the eligibility criteria, and the sources and methods of selection of participants. Describe methods of follow-up.	4
Eligibility criteria	#6b	For matched studies, give matching criteria and number of exposed and unexposed	4
Variables	#7	Clearly define all outcomes, exposures, predictors, potential confounders, and effect modifiers. Give diagnostic criteria, if applicable	6
Data sources / measurement	#8	For each variable of interest give sources of data and details of methods of assessment	66

For peer review only

		(measurement). Describe comparability of assessment methods if there is more than one group. Give information separately for for exposed and unexposed groups if applicable.	
Bias	#9	Describe any efforts to address potential sources of bias	6
Study size	#10	Explain how the study size was arrived at	4
Quantitative variables	#11	Explain how quantitative variables were handled in the analyses. If applicable, describe which groupings were chosen, and why	6
Statistical methods	#12a	Describe all statistical methods, including those used to control for confounding	
6,7			
Statistical methods	#12b	Describe any methods used to examine subgroups and interactions	7
Statistical methods	#12c	Explain how missing data were addressed	7
Statistical methods	#12d	If applicable, explain how loss to follow-up was addressed	NA
Statistical methods	#12e	Describe any sensitivity analyses	
7			

For peer review only

Results

Participants	#13a	Report numbers of individuals at each stage of study—eg numbers potentially eligible, examined for eligibility, confirmed eligible, included in the study, completing follow-up, and analysed. Give information separately for for exposed and unexposed groups if applicable.	Figure 1
Participants	#13b	Give reasons for non-participation at each stage	Figure 1
Participants	#13c	Consider use of a flow diagram	
Figure 1			
Descriptive data	#14a	Give characteristics of study participants (eg demographic, clinical, social) and information on exposures and potential confounders. Give information separately for exposed and unexposed groups if applicable.	Table 1
Descriptive data	#14b	Indicate number of participants with missing data for each variable of interest	
16			
Descriptive data	#14c	Summarise follow-up time (eg, average and total amount)	
5			
Outcome data	#15	Report numbers of outcome events or summary	

For peer review only

		measures over time. Give information separately for exposed and unexposed groups if applicable.	
17-19			
Main results	#16a	Give unadjusted estimates and, if applicable, confounder-adjusted estimates and their precision (eg, 95% confidence interval). Make clear which confounders were adjusted for and why they were included	8, 9, 17-19
Main results	#16b	Report category boundaries when continuous variables were categorized	6
Main results	#16c	If relevant, consider translating estimates of relative risk into absolute risk for a meaningful time period	9, 18
17-18			
Other analyses	#17	Report other analyses done—eg analyses of subgroups and interactions, and sensitivity analyses	8,9, Supplemental figures 2 & 3
**Discussion**			
Key results	#18	Summarise key results with reference to study objectives	9
Limitations	#19	Discuss limitations of the study, taking into account sources of potential bias or imprecision. Discuss both direction and magnitude of any potential bias.	10
Interpretation	#20	Give a cautious overall interpretation considering	11

For peer review only

		objectives, limitations, multiplicity of analyses, results from similar studies, and other relevant evidence.	
Generalisability	#21	Discuss the generalisability (external validity) of the study results	10
**Other Information**			
Funding	#22	Give the source of funding and the role of the funders for the present study and, if applicable, for the original study on which the present article is based	12

Notes:

- 16a: 8, 9, 17-19
- 17: 8,9, Supplemental figures 2 & 3 The STROBE checklist is distributed under the terms of the Creative Commons Attribution License CC-BY. This checklist was completed on 21. January 2022 using https://www.goodreports.org/, a tool made by the EQUATOR Network in collaboration with Penelope.ai

For peer review only
